# Performance validation of deep-learning-based approach in stool examination

**DOI:** 10.1186/s13071-025-06878-w

**Published:** 2025-08-01

**Authors:** Kristal Dale Felimon Corpuz, Teera Kusolsuk, Benjamaporn Wongphan, Putza Chonsawat, Kaung Myat Naing, Siridech Boonsang, Veerayuth Kittichai, Chia-Kwung Fan, Santhad Chuwongin, Dorn Watthanakulpanich

**Affiliations:** 1 Department of Helminthology, Faculty of Tropical Medicine, Mahidol University, Bangkok, Thailand; 2 Hospital for Tropical Diseases, Faculty of Tropical Medicine, Mahidol University, Bangkok, Thailand; 3 Center of Industrial Robot and Automation (CIRA), College of Advanced Manufacturing Innovation, King Mongkut’s Institute of Technology Ladkrabang, Bangkok, Thailand; 4 Department of Electrical Engineering, School of Engineering, King Mongkut’s Institute of Technology Ladkrabang, Bangkok, Thailand; 5 Faculty of Medicine, King Mongkut’s Institute of Technology Ladkrabang, Bangkok, Thailand; 6 Department of Molecular Parasitology and Tropical Diseases, School of Medicine, College of Medicine, Taipei Medical University, Taipei, Taiwan; 7 Department of Biological Sciences, College of Science, Central Luzon State University, Nueva Ecija, Philippines

**Keywords:** Intestinal parasitic infection, Self-supervised learning, Deep learning, Automation

## Abstract

**Background:**

Human intestinal parasitic infections (IPI) pose a significant global health issue caused by parasitic helminths and protozoa, affecting around 3.5 billion people worldwide, with more than 200,000 deaths annually. Despite advancements in molecular methods with higher sensitivity and specificity, the Kato-Katz or formalin-ethyl acetate centrifugation technique (FECT) remains the gold standard and a routine diagnostic procedure suitable for its simplicity and cost-effectiveness. However, these techniques have limitations that must be addressed. Thus, this study evaluated the performance of a deep-learning-based approach for intestinal parasite identification and compared it with that of human experts.

**Methods:**

Human experts performed FECT and Merthiolate-iodine-formalin (MIF) techniques to serve as ground truth and reference for parasite species. Subsequently, a modified direct smear was conducted to gather images for the training (80%) and testing (20%) datasets. State-of-the-art models, including YOLOv4-tiny, YOLOv7-tiny, YOLOv8-m, ResNet-50, and DINOv2 (base, small, and large), were employed and were operated using in-house CIRA CORE platform. Overall performance was evaluated using confusion matrices, the metrics of which were calculated on the basis of the one-versus-rest and micro-averaging approaches. Moreover, the receiver operating characteristic (ROC) and precision-recall (PR) curves were determined for visual comparison. Lastly, Cohen’s Kappa and Bland–Altman analyses were used to statistically measure the significant differences and visualize the association levels between the human experts and the deep learning models’ classification performance in intestinal parasite identification.

**Results:**

Findings demonstrated the potential of a deep-learning-based approach, particularly of models DINOv2-large (accuracy: 98.93%; precision: 84.52%; sensitivity: 78.00%; specificity: 99.57%; F1 score: 81.13%; AUROC: 0.97) and YOLOv8-m (accuracy: 97.59%; precision: 62.02%; sensitivity: 46.78%; specificity: 99.13%; F1 score: 53.33%; AUROC: 0.755; AUPR: 0.556) for their high metric values in intestinal parasite identification. Class-wise prediction showed high precision, sensitivity, and F1 scores for helminthic eggs and larvae due to more distinct morphology. Moreover, all models obtained a > 0.90 k score, which indicates a strong level of agreement compared with the medical technologists. The Bland–Altman analysis also presented the best agreement between FECT performed by medical technologist A and YOLOv4-tiny, while the MIF technique performed by medical technologist B and DINOv2-small demonstrated the best bias-free agreement, with mean differences of 0.0199 and −0.0080, and standard deviation differences of 0.6012 and 0.5588, respectively.

**Conclusions:**

The results highlight the potential of integrating a deep-learning-based approach into parasite identification. The models showcased superiority in automated detection, suggesting a significant leap toward improving diagnostic procedures for IPI. This hybridization could enhance early detection and diagnosis, facilitating timely and targeted interventions to reduce the burden of IPI through more effective management and prevention strategies.

**Graphical Abstract:**

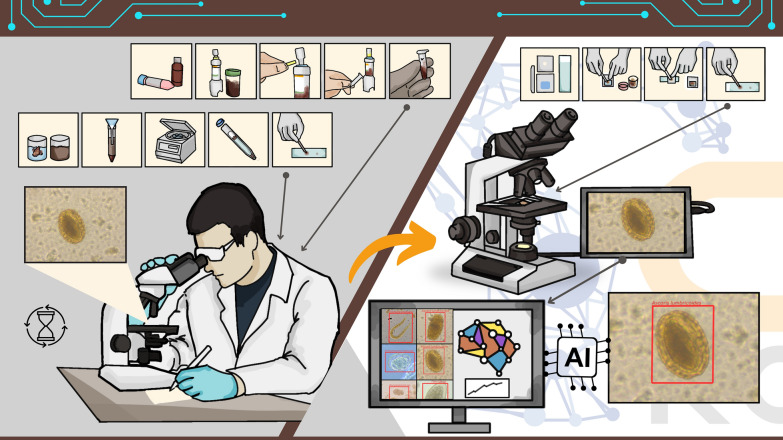

## Background

Human intestinal parasitic infections (IPI), primarily caused by helminths and protozoans, are prevalent in underprivileged communities and developing countries. Prevalence varies in rural and urban settings, with even sporadic cases reported in developed countries. Meanwhile, vulnerable areas face higher risks due to poor water supply, sanitation, and hygiene. Several studies have assessed impact of IPI on public health, revealing regional variations in disease burden influenced by environmental, socioeconomic, and cultural factors. Global estimates indicate 819 million cases of *Ascaris lumbricoides*, 464 million of *Trichuris trichiura*, and 438 million of hookworms [1], with approximately 352 million infections attributed to parasitic protozoans [2].

In macroscopic examination, adult ascarids and pinworms are morphologically identified on the basis of size, shape, and color, while tapeworm species are recognized through their proglottids. Meanwhile, smaller parasitic products, such as eggs, larvae, cysts or oocysts, and trophozoites, require microscopic examination. Kato-Katz and other coprological techniques remain the gold standard for routine diagnostic procedures due to their simplicity and cost-effectiveness [3]. Direct smears provide a rapid and cost-effective definitive diagnosis for primary assessment or field investigations. However, these techniques’ qualitative and quantitative performance is unsuitable for multiple infections, especially in endemic areas.

Some procedures based on centrifugation, floatation, and culture techniques account for the distinct characteristics of target parasites and the intensity of parasitic load from samples to maximize detection. The formalin-ether concentration technique (FECT) was introduced in the 1940 s, involving mixing stool samples with a formalin-ether solution followed by centrifugation to improve the detection of low-level infections [4]. FECT—later switched ether to ethyl acetate, used at the Centers for Disease Control and Prevention—is suitable for examining preserved stool samples, yet results may vary on the basis of the analyst [5].

Another persisting challenge is the smaller sizes and shared morphology for protozoans, but staining techniques are advantageous for differentiating species and enhancing visibility by providing better contrast. The Merthiolate-iodine-formalin (MIF) technique is an effective fixation and staining solution with easy preparation and long shelf life, making it suitable for field surveys. Research by Incani et al. indicates that MIF addresses the practical drawbacks of direct stool examination and provides highly competitive performance for evaluating IPI [6]. However, limitations include incompatibility with certain trichrome stains, inadequate preservation of trophozoite morphology, and potential distortion due to iodine, necessitating careful consideration in research applications [7].

Molecular techniques have become valuable diagnostic tools for detecting parasitic biomolecules in stool samples, offering greater sensitivity and specificity than conventional methods. Polymerase chain reaction enhances diagnostic accuracy [8], but effective deoxyribonucleic acid extraction is essential for optimal results [9]. Despite their advantages, molecular methods are often time-consuming and costly, require skilled personnel, and carry contamination risks [10]. Addressing these challenges, artificial intelligence presents a promising future for diagnostics through the application of machine learning and deep-learning-based algorithms using advanced image analysis, pattern recognition, and feature extraction. Machine learning applies algorithms and statistical models for efficient data management, while deep learning excels at processing complex data, such as images, genetic sequences, and epidemiological input [11].

In the classification approach, neural networks categorize input datasets by learning features and distinct patterns, enabling accurate identification of unseen data. Li et al. introduced the FecalNet method on the basis of RetinaNet, achieving a mean average precision (mAP) of 92.16% and an average recall (AR) of 93.56% for various parasitic eggs, including hookworm (AP 89.95%, AR 93.88%), *A. lumbricoides* (AP 96.90%, AR 91.21%), and *T. trichiura* (AP 88.61%, AR 94.37%) [12, 13]. In another study, Reddy and Juliet proposed transfer learning to improve diagnosis accuracy for malaria using ResNet-50 on 27,558 cell images, achieving 95.91% training and 95.4% validation accuracy [14]. Moreover, Zhu et al. developed ROENet based on ResNet-18 for classifying *Plasmodium* spp. in blood smears through fivefold cross-validation, obtaining 96.68%, 94.79%, 95.73%, and 95.69% for specificity, sensitivity, accuracy, and F1 score, respectively [15].

In contrast, the object detection approach uses algorithms to enhance the identification of parasitic products. Redmon et al. reframed object detection as a single regression problem directly from image pixels to bounding boxes and class probabilities, streamlining the process compared with previous systems that required multiple evaluations [16–18]. A trained convolutional neural network (CNN) achieves a high recognition performance for parasitic eggs, while object detection models showcase potential in more accurate parasitic identification, especially for mixed infections [19, 20]. Recently, the You Only Look Once (YOLO) one-stage detection model gained popularity for detecting multiple objects in an image. With relevance to parasitology, Naing et al. conducted a comparative study of YOLO models (YOLOv4-tiny, YOLOv3, and YOLOv3-tiny) to automatically recognize 34 classes of parasites, finding YOLOv4-tiny superior with 96.25% precision, 95.08% sensitivity, and the highest area under the precision-recall curve (AUPRC) score of 0.963 [21].

However, the abovementioned deep-learning-based models require manually labeled datasets, which decelerate the process and are often impractical for large datasets. To overcome this, self-supervised learning (SSL) utilize features from unlabeled datasets [22]. A notable model, Distillation of Knowledge with NO Labels (DINO)v2, employs Vision Transformers (ViT) for image recognition, learning features independently even with limited images. Oquab et al.’s benchmark data indicate that DINOv2 outperforms existing all-purpose features, making it favorable for computer vision applications. DINOv2 benefits from hyperparameter tuning and a unique data curation pipeline to enhance training speed and stability [23]. Pinetsuksai et al. successfully screened common human helminth eggs in Thailand—*A. lumbricoides*, hookworm, *C. philippinensis*, *E. vermicularis*, *F. buski*, *H. diminuta*, *H. nana*, *O. viverrini*, *Paragonimus* spp., *Taenia* spp., and *T. trichiura* —using an online network for feature extraction without class labeling and data-clustering-based similarity loss function. Findings demonstrated that the advanced bootstrap your own latent (BYOL) method is effective even with limited data resources (1–10%). DINOv2-distilled models were further trained to automatically screen helminth infection using a large and curated dataset designed on ViT architecture, employing a sequential classifier that transforms data to 256 dimensions before mapping it to class numbers. DINOv2 models were developed as ViT-S (small), ViT-B (base), and ViT-L (large), and compared with BYOL at 1–10% dataset fractions to identify the most efficient approach. Despite tradeoffs between data fraction and efficiency, results showed DINOv2-L as the best model at a 10% data fraction, achieving 99.0% accuracy, 93.7% recall, 95.2% precision, 99.9% specificity, 94.3% F1 score, and 99.0% AUC [24].

Given these developments, this study aimed to evaluate the effectiveness and performance of selected SL and SSL-based deep learning models for identifying human intestinal parasites from stool samples. Specifically, the assessment of classification (ResNet-50), object detection (YOLOv4-tiny, YOLOv7-tiny, YOLOv8-m), and DINOv2 models using a well-curated dataset of microscopic images containing a variation of parasitic products.

## Methods

**Fig 1.**
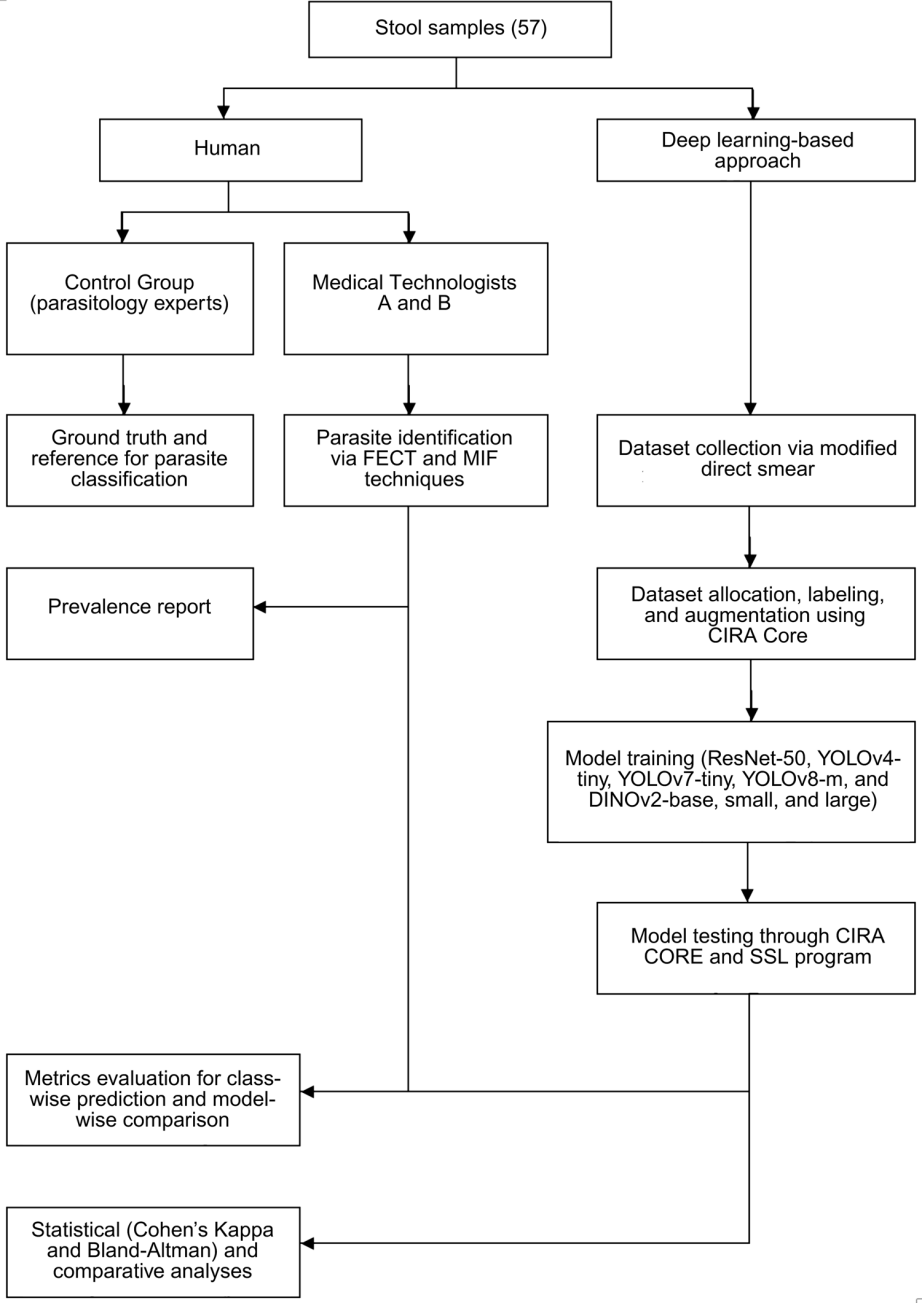
Study design for the general scheme followed to accomplish the objectives of the study

The general scheme of this study (Fig. 1) involved the diagnosis of 57 stool samples using the FECT and MIF techniques by two parasitology experts (control) from the Faculty of Tropical Medicine, Mahidol University. Additionally, two medical technologists from the Hospital for Tropical Medicine, Mahidol University, conducted blinded stool examinations of the same samples. It was followed by model training and testing utilizing parasitic product images obtained through a modified direct smear technique.

### Conventional stool examination

Human experts in parasitology served as the control group, while medical technologists A and B represented human-based stool examinations compared with selected deep-learning-based models. Each medical technologist examined 57 leftover stool samples from Tak Province, Northern Thailand, stored for teaching and laboratory practice at the Department of Helminthology, Faculty of Tropical Medicine, Mahidol University, using conventional methods such as the FECT and MIF techniques.

**Fig 2.**
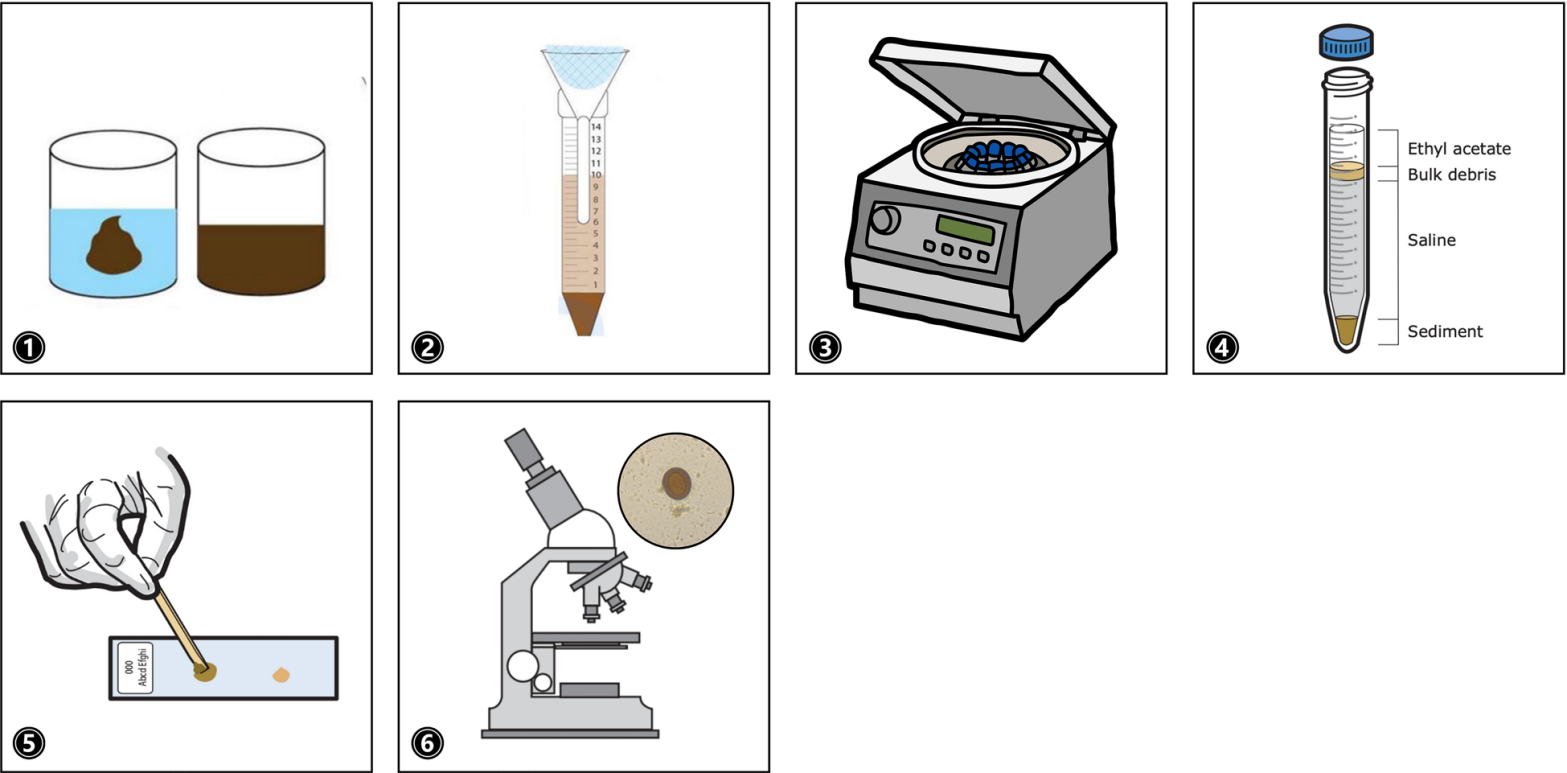
Illustration of the FECT as a key conventional method for concentrating parasitic products prior to microscopic examination [25]: (**1**) mix the stool sample with NSS; (**2**) use wet gauze to strain into a centrifugal tube and (**3**) centrifuge at 2000 rpm; decant supernatant and add the formalin, mix, then add the ethyl acetate and centrifuge again; (**4**) observe separation of solution into layers including the sediment; (**5**) prepare a slide from the precipitate; and (**6**) observe under a microscope. Images adapted from the World Health Organization (WHO; 2019)

FECT is recommended as a simple and effective procedure for recovering protozoan cysts, helminthic eggs, and larval parasites (Fig. 2). Its principle relies on the extraction of fecal constituents and utilizing ethyl acetate to dissolve fats, formalin to preserve the integrity of structures, and centrifugation to enhance sedimentation: (1) 1–2 g (g) of stool sample was added into 10 ml (mL) of normal saline solution and mix well; (2) over a funnel, two layers of wet gauze were used to strain the solution into a 15 mL centrifugal tube and (3) centrifuged at 2000 rpm for 2 min; decant the supernatant and 8 mL of 10% formalin solution was added into the sediment, mixed well, and left to stand for 5 min; 3 mL of ethyl acetate was then added, cap closed tightly, tube shaken vigorously, and again centrifuged for 2 min at 2000 rpm; (4) after centrifugation, the solution was divided into four layers composed of the ethyl acetate and fat, debris plug, formalin, and the sediment at the bottom; to loosen the plug between the layers of the formalin and ethyl acetate, a stick was inserted between the tube wall and fecal plug and moved in a circular motion while holding the tube horizontally; before going back to vertical position, a piece of cotton was used to clean the sides of the tube to prevent contaminating the precipitate; (5) a portion of the precipitate was drawn using a pipette and a drop put onto a glass slide, covered with a cover slip; and (6) observed under the microscope [25].

**Fig 3.**
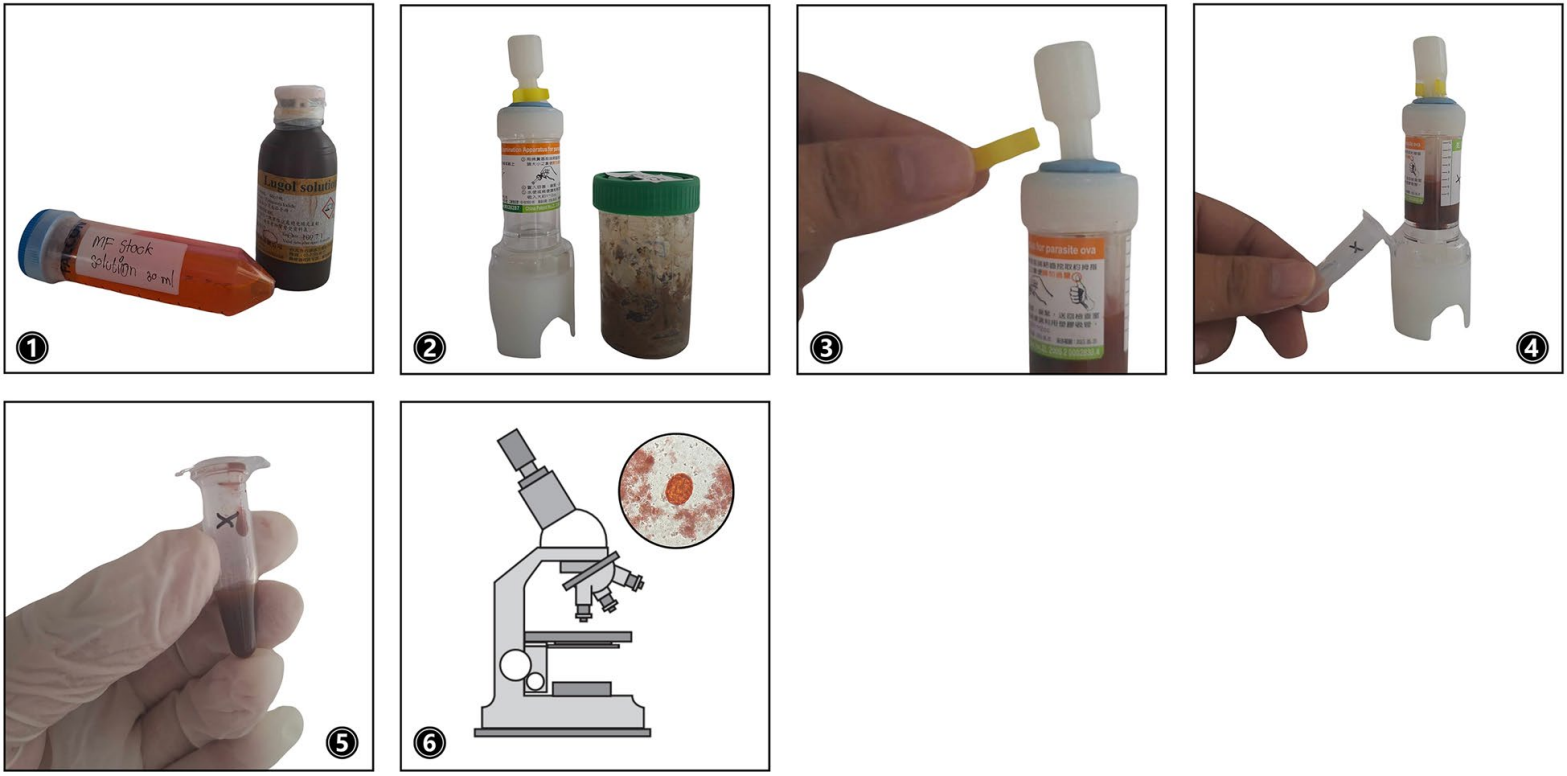
Illustration of the MIF technique performed to involve staining and preservation of parasitic products for enhanced microscopic observation [26]: (**1**) prepare the MIF solution in 15:1 ratio; (**2**) add the stool sample to a Chang’s container, mix with the MIF solution, and let stand; (**3**) after 2 h, remove the clip to push the stirrer through the bottom enabling filtration; (**4**, **5**) collect the filtrate into a centrifugal tube; and (**6**) place a drop of the filtrate onto a glass slide and observe under the microscope. Images captured by the main author

The MIF technique was complemented with Chang’s feces examination apparatus, which enhances morphological fixation and staining (Fig. 3): (1) MF stock solution and Lugol’s solution were mixed in a 15:1 ratio to prepare the MIF stock solution; (2) 1 g of stool sample was placed into Chang’s container, to which 5–10 mL of the MIF solution was added and allowed to stand for at least 2 h; (3) then the clip was removed from the container, enabling the attached stirrer on the lid to be pushed through the bottom, thus filtering the solution; (4) the filtrate was collected in an appropriately sized polypropylene centrifugal tube; (5) a drop was placed onto a glass slide, covered with a cover slip; and (6) examined under a microscope [26].

### Dataset collection and allocation

**Fig 4.**
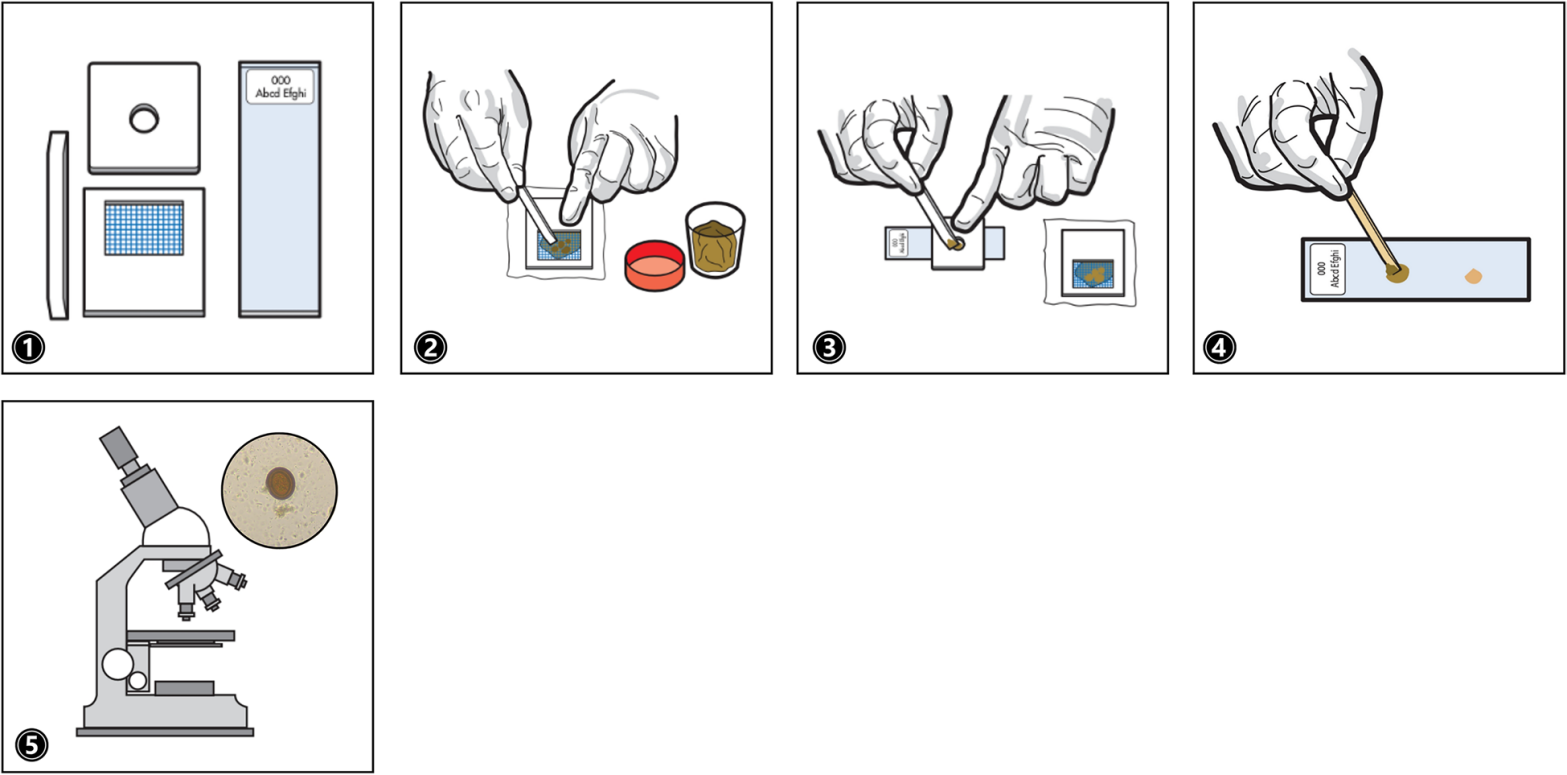
Illustration of the modified direct smear performed to collect dataset images [21]: (**1**) prepare the material needed; (**2**) an adequate amount of stool sample is pressed through a mesh screen to isolate a portion; (**3**) transfer the portion into the hole of a cardboard template placed onto a glass slide; (**4**) place a drop of NSS and/or Lugol’s iodine solution onto the sample and mix well; and (**5**) cover the specimen with cover slip and observe under the microscope. Images adapted from the WHO (2019)

Subsequently, the same stool samples were screened via modified direct smear (on the basis ofKato-Katz) to photo-document the parasites present and were compiled as datasets composed of 34 classes (Fig. 4): (1) all the materials needed were prepared including the 105-mesh screen, a cardboard template (3 × 4 × 0.137 cm), stool samples, applicator sticks, 0.85% normal saline solution (NSS), 1% Lugol’s iodine solution, glass slides, and cover slips (22 × 30 cm); (2) an adequate amount of stool was pressed through the mesh screen to remove large particles and debris; (3) the isolated portion was then transferred into the hole of a cardboard template placed on a glass slide; (4) a drop of 0.85% normal saline solution (NSS) was added to the sample, stirred, and covered with a coverslip; for nonspecific contrast to facilitate the identification of protozoans, a drop of 1% Lugol’s iodine solution was also applied; (5) the prepared slides were examined under a microscope using 10× to screen for suspected parasites; 40× objective lens was used for final magnification, and images were photo-documented using an Axiocam Camera embedded in a ZEISS Primo Vert Microscope, connected to Zen 2.3 (blue edition) software on a Windows 10 installed ACER desktop computer with set dimensions of 2560 × 1920 pixels, 24-bit depth, and as JPG file format [21].

**Fig 5.**
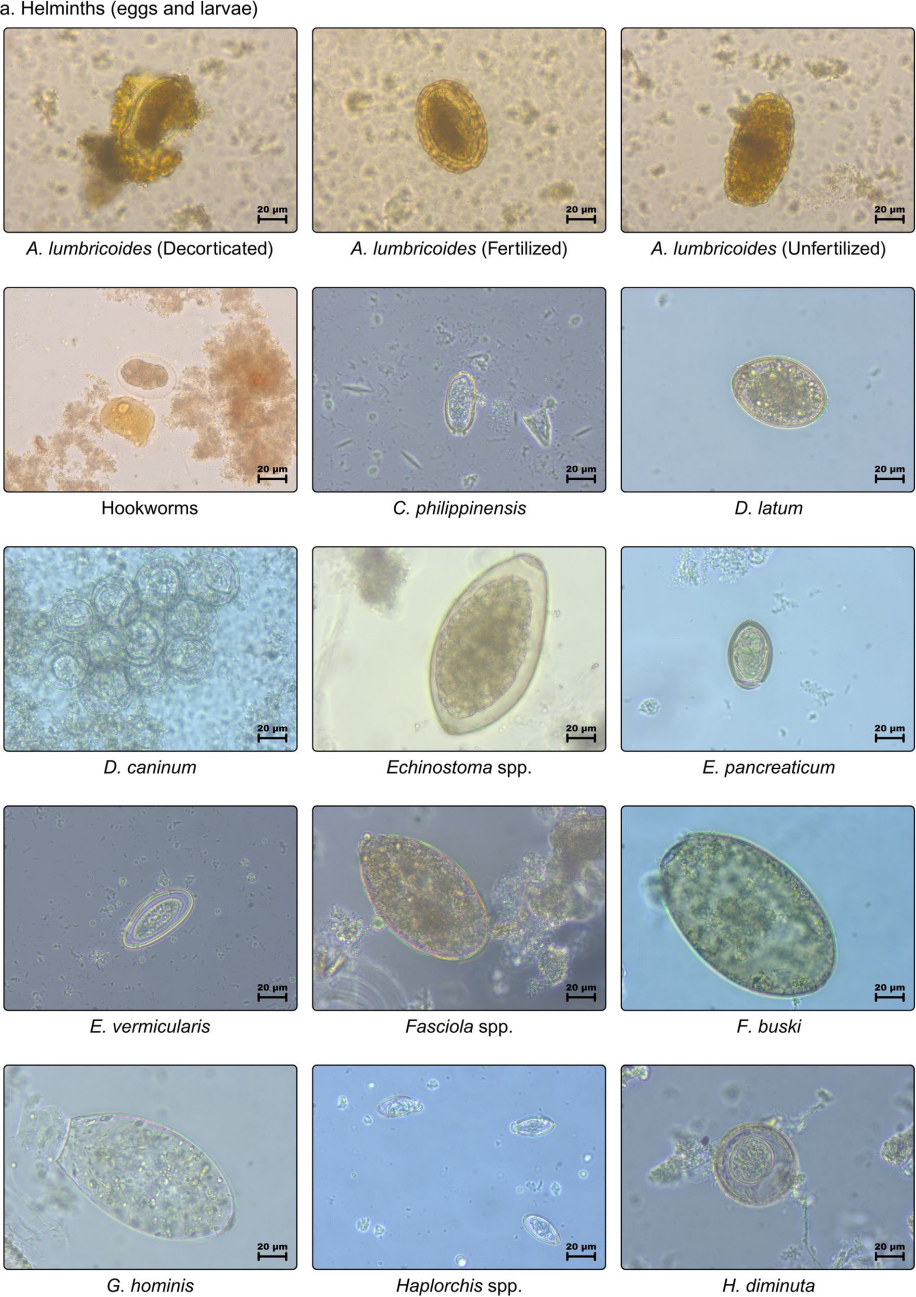

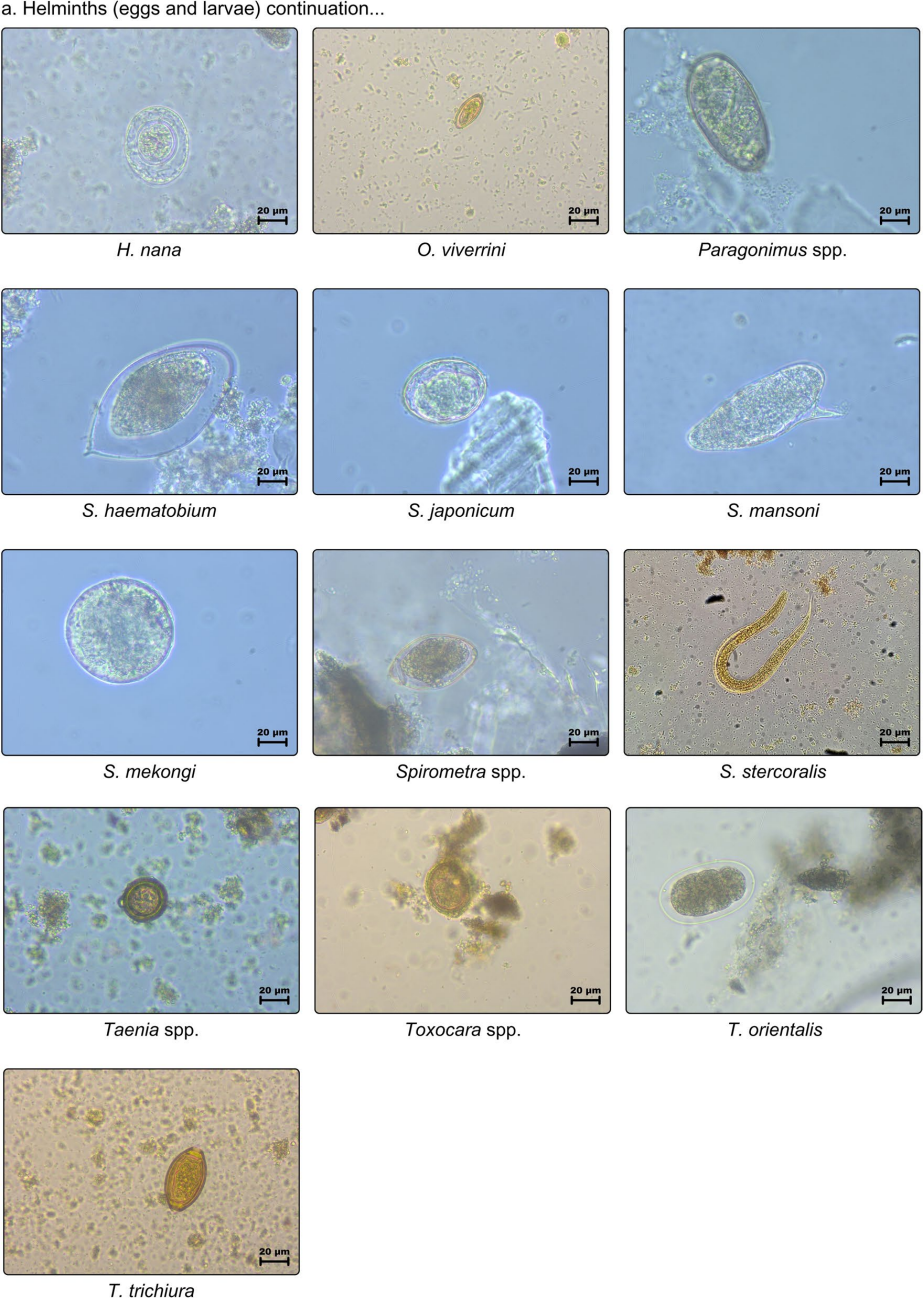

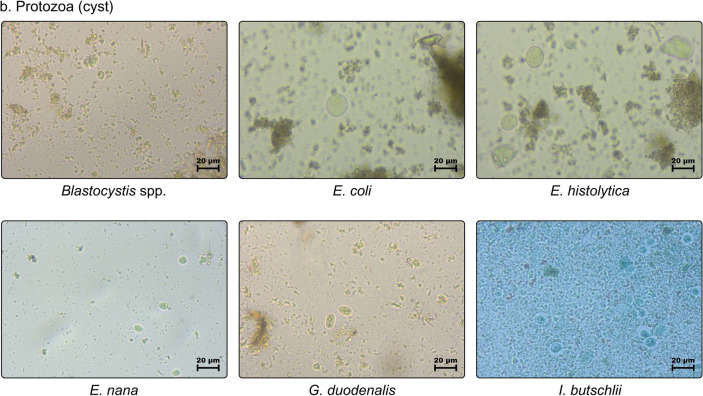
Training dataset consisted of 34 classes of unlabeled parasite images (40×) of helminthic and protozoan species: (**a**) helminths (eggs and larvae), and (**b**) protozoa (cyst)

Datasets (Table 1 and Fig. 5) are composed of 28 helminths and six protozoans, with 2567 images allocated as 80% (2054) for training and 20% (513) for testing. Although there is no defined proportion to the number of images for datasets, this component can influence the success rate of the process. Therefore, it should be more than 50%. These images were adapted from Naing et al., with additional images from the recently examined 57 stool samples dedicated to this study. However, only 14 classes with 343 images were preferred as testing datasets solely obtained from the new stool samples to assume the difference of field settings for real-time detection and using a pre-prepared training dataset, wherein models were trained with various classes and then the best-fit model was used to identify observed parasites.

**Table 1:** Quantity of images per parasite class used as training and testing datasets

Classes (34)	Abbr	Total # of images	Training (80%)	Testing (20%)
Protozoa				
*Blastocystis* spp.	BLS	61	49	12*
*Entamoeba coli*	ENC	227	182	45*
*Entamoeba histolytica*	ENH	80	64	16*
*Endolimax nana*	ENN	141	113	28*
*Giardia duodenalis*	GID	371	297	74*
*Iodamoeba butschlii*	IOB	40	32	8
Helminths				
*Ascaris lumbricoides* (decorticated)	ALD	45	36	9*
*Ascaris lumbricoides* (fertilized)	ALF	393	314	79*
*Ascaris lumbricoides* (unfertilized)	ALU	31	25	6*
*Capillaria philippinensis*	CAP	30	24	6
*Dipylidium caninum*	DIC	56	45	11
*Diphyllobothrium latum*	DIL	31	25	6
*Eurytrema pancreaticum*	EUP	23	18	5
* Enterobius vermicularis*	ENV	30	24	6
* Echinostoma* spp.	ECS	25	20	5
*Fasciolopsis buski*	FAB	65	52	13
* Fasciola* spp.	FAS	28	22	6
*Gastrodiscoides hominis*	GAH	40	32	8
*Hymenolepis diminuta*	HYD	48	38	10
*Hymenolepis nana*	HYN	26	21	5
*Haplorchis* spp.	HAS	35	28	7
Hookworms	HKW	35	28	7*
*Opistorchis viverrini*	OPV	51	41	10*
*Paragonimus* spp.	PAS	109	87	22
*Schistosoma haematobium*	SCH	31	25	6
*Schistosoma japonicum*	SCJ	22	18	4
*Schistosoma mansoni*	SMA	80	64	16
* Schistosoma mekongi*	SME	61	49	12
*Strongyloides stercoralis*	STS	43	34	9*
*Spirometra* spp.	SPS	54	43	11
*Trichostrongylus orientalis*	TRO	16	13	3
*Trichiuris trichiura*	TRT	37	30	7*
*Taenia* spp.	TAS	133	106	27*
*Toxocara* spp.	TOS	69	55	14*
Total		2,567	2,054	513

^*^Images included in testing dataset

### Model selection, training, and testing

State-of-the-art SL and SSL models were selected and employed for training and testing, specifically ResNet-50 (2015), YOLOv4-tiny (2020), YOLOv7-tiny (2022), YOLOv8-m (2023), and DINOv2 (2023). The YOLO series, known for its efficiency in real-time object detection, predicts bounding boxes and class probabilities effectively. ResNet-50 offers superior performance in image classification, while DINOv2 facilitates automatic annotation by leveraging unlabeled datasets, making it suitable for large datasets.

Model training utilized a consistent dataset across all models. YOLO models underwent manual labeling via the *DeepTrain* function, whereas ResNet-50 and DINOv2 utilized unlabeled data. To enhance dataset diversity and ensure accuracy, augmentation techniques were applied for both training and testing datasets: for training (rotation: −180–180°, contrast: 0.4–1.1, blur: 9, noise: 30) and for testing (rotation: 0–180°, contrast: 0.4–1.1, blur: 9, noise: 30). During testing, models predicted class labels on previously unseen images, ensuring varied input across training and testing phases. YOLO models employed node-flow programming for continuous testing and image recognition; ResNet-50 utilized the *EvalClassif* function; and DINOv2 variants were tested via CIRA cloud integration with SSL programs. CIRA Robotics executed all procedures on the CIRA CORE platform [https://git.cira-lab.com/cira/cira-core], which supports AI-related tasks, including object detection and classification [21, 27].

### Performance and metrics evaluation

The performance evaluation used confusion matrices to interpret results as true positives (TP: number of parasites correctly classified as positive), true negatives (TN: number of parasites correctly classified as negative), false positives (FP: number of parasites incorrectly classified as positive), and false negatives (FN: number of parasites incorrectly classified as negative) derived from model testing. These metrics allowed for the calculation of various performance metrics: (*i*) accuracy refers to the ratio between the correctly classified parasites and the total number of images in the testing dataset; (*ii*) precision is the proportion of relevant parasites from the ratio of correctly classified parasites and all predictions made for the class; (*iii.*) sensitivity denotes the rate of correct positive classifications from the ratio of correctly classified positives and all the images assigned as positive; (*iv*) specificity is the rate of correct negative classifications from the ratio of correctly classified negatives and all the images assigned as negative, and (*v*) F1 score is the harmonic mean of precision and sensitivity. With assumed multiple classes, a one-versus-rest approach and micro-averaging calculations were used for adaptations in calculations considering multiple-label classification [28, 29].

$$Accuracy=\frac{{\sum }_{i=1}^{n}TPi+TNi}{{\sum }_{i=1}^{n}TPi+FPi+TNi+FNi}$$

$$Precision=\frac{{\sum }_{i=1}^{n}TPi}{{\sum }_{i=1}^{n}TPi+FPi}$$

$$Sensitivity=\frac{{\sum }_{i=1}^{n}TPi}{{\sum }_{i=1}^{n}TPi+FNi}$$

$$Specificity=\frac{{\sum }_{i=1}^{n}TNi}{{\sum }_{i=1}^{n}TNi+FPi}$$

$$F1 Score= 2\left(\frac{Precison\, x\, Sensitivity}{Precision+Sensitivity}\right)$$

The receiver operating characteristic (ROC) and precision-recall (PR) curves were analyzed to evaluate overall model performance, represented by the area under the ROC (AUROC) and AUPRC. The ROC illustrates how correctly classified positives relate to incorrectly classified negatives, ideally approaching the upper left-hand corner with a maximum value of 1. Conversely, the PR curve displays precision and sensitivity across confidence thresholds, particularly useful in multilabel classification comparisons [30]. Additionally, the uniform manifold approximation and projection (UMAP) visualization was included for DINOv2, illustrating the uniform distribution of data points and highlighting the algorithm’s effectiveness [31].

These metrics provide quantitative and objective measures to assess model performance, guide optimization techniques, and serve as a benchmarking tool in deep learning and parasitology, enhancing understanding of model behavior and performance.

### Statistical analysis

Cohen’s kappa measures the significant differences in the association levels between human performance and deep learning approaches [32]. It assesses interrater reliability by comparing agreement levels across methods, using both observed and coincidence agreement, with a statistical range from −1 to 1 based on counts of TP, TN, FP, and FN by medical technologists and the model. The Kappa score (*κ*) was calculated, and interpretations from McHugh (2012) provided guidance on the final level of agreement [33]. Moreover, Bland–Altman analysis was conducted to compare per-class agreement and overall performance between humans and the models. Both analyses were performed using RStudio [https://cran.r-project.org/].

## Results

### Conventional stool examination and parasite identification

A total of 11 classes were identified using FECT and MIF techniques by medical technologists, with 5 helminths and 4 protozoan species detected among the 57 examined samples—*A. lumbricoides* (decorticated, fertilized, and unfertilized), hookworms, *O. viverrini*, *Taenia* spp., *T. trichiura*, *E. coli*, *E. histolytica*, *E. nana*, and *G. duodenalis*—consistent with routine laboratory findings for parasitic protozoans and notorious STH. To consolidate the detections from both analysts in FECT and MIF techniques and produce a single prevalence estimate, a *t*-test confirmed no significant differences in counts, which were then averaged for percent prevalence calculation (Table 2). *E. coli* and fertilized *A. lumbricoides* were the most prevalent parasites, while decorticated *A. lumbricoides*, hookworms, and *O. viverrini* had the lowest prevalence. Compared with control experts, no current infections were detected for *Blastocystis* spp., *E. vermicularis*, and *S. stercoralis*.

**Table 2:** Prevalence report gathered from conventional stool examination

Class (species)	FECT_avg	% Prev	MIF_avg	% Prev
*A. lumbricoides* (decorticated)	0.50	0.88	0.00	0.00
*A. lumbricoides* (fertilized)	11.00	19.30	10.50	18.42
*A. lumbricoides* (unfertilized)	3.00	5.26	3.00	5.26
Hookworms	2.00	3.51	0.50	0.88
*Blastocystis* spp.	1.00	1.75	1.00	1.75
*E. coli*	18.5	32.46	18.00	31.58
*E. histolytica*	3.00	5.26	3.00	5.26
*E. nana*	3.50	6.14	4.50	7.89
*G. duodenalis*	2.00	3.51	2.00	3.51
*O. viverrini*	0.50	0.88	1.00	1.75
*Taenia* spp.	3.00	5.26	3.00	5.26
*T. trichuria*	9.00	15.79	3.00	5.26

Note: *avg* average detection count per class for each method, *% Prev* percent prevalence

For class-wise comparison of parasites identified by medical technologists, TP, TN, FP, and FN counts were established relative to control expert findings, allowing for the calculation of precision, sensitivity, and F1 scores (Tables 3 and 4). Both analysts exhibited high metrics for *G. duodenalis* and *O. viverrini* in both techniques, while consistent values were noted for fertilized and unfertilized *A. lumbricoides*, *E. coli*, and *Taenia* spp. Variations were observed in the analysts’ detection concerning decorticated *A. lumbricoides* and hookworms in FECT, with overall performance metrics exceeding 44%. However, null values were recorded for decorticated and unfertilized *A. lumbricoides*, *Blastocystis* spp., *E. vermicularis*, hookworms, *S. stercoralis*, and *T. trichiura* due to FP and FN counts.

**Table 3:** Class-wise comparison of metrics (in percentage) among the parasites detected via FECT

Class (species)	FECT Med Tech A			FECT Med Tech B		
	Prec	Sen	F1	Prec	Sen	F1
*A. lumbricoides* (decorticated)	0.00	0.00	0.00	100.00	50.00	66.67
*A. lumbricoides* (fertilized)	50.00	85.71	63.16	66.67	85.71	75.00
*A. lumbricoides* (unfertilized)	100.00	40.00	57.14	100.00	40.00	57.14
*Blastocystis* spp.	0.00	0.00	0.00	0.00	0.00	0.00
*E. coli*	66.67	75.00	70.59	66.67	75.00	70.59
*E. histolytica*	50.00	100.00	66.67	50.00	50.00	50.00
*E. nana*	83.33	83.33	83.33	66.67	33.33	44.44
*E. vermicularis*	0.00	0.00	0.00	0.00	0.00	0.00
*G. duodenalis*	100.00	100.00	100.00	100.00	100.00	100.00
Hookworms	66.67	100.00	80.00	0.00	0.00	0.00
*O. viverrini*	100.00	100.00	100.00	100.00	100.00	100.00
*S. stercoralis*	0.00	0.00	0.00	0.00	0.00	0.00
*Taenia* spp.	100.00	75.00	85.71	100.00	75.00	85.71
*T. trichuria*	41.67	62.50	50.00	50.00	12.50	20.00

Notes: *Prec* precision, *Sen* sensitivity, *F1* F1 score, *Med Tech* Medical Technologist

**Table 4:** Class-wise comparison of metrics among the parasites detected via MIF technique

Class (species)	MIF Med Tech A			MIF Med Tech B		
	Prec	Sen	F1	Prec	Sen	F1
*A. lumbricoides* (decorticated)	0.00	0.00	0.00	0.00	0.00	0.00
*A. lumbricoides* (fertilized)	81.82	90.00	85.71	81.82	90.00	85.71
*A. lumbricoides* (unfertilized)	0.00	0.00	0.00	0.00	0.00	0.00
*Blastocystis* spp.	0.00	0.00	0.00	0.00	0.00	0.00
*E. coli*	71.43	71.43	71.43	85.71	85.71	85.71
*E. histolytica*	69.00	28.57	44.44	100.00	42.86	60.00
*E. nana*	60.00	60.00	60.00	66.67	80.00	72.73
Hookworms	0.00	0.00	0.00	0.00	0.00	0.00
*O. viverrini*	100.00	100.00	100.00	100.00	100.00	100.00
*Taenia* spp.	33.33	100.00	50.00	33.33	100.00	50.00
*T. trichuria*	0.00	0.00	0.00	0.00	0.00	0.00

Notes: *Prec* precision, *Sen* sensitivity, *F1* F1 score, *Med Tech* Medical Technologist

The evaluation of both medical technologists using FECT and MIF techniques is summarized in Table 5. Medical Technologist A showed superior sensitivity in recognizing TPs with FECT, while Medical Technologist B demonstrated better specificity for negative results. Both analysts achieved high accuracy (above 94%) and precision (above 63%), with Medical Technologist B performing better in the MIF technique. Overall, Medical Technologist A achieved the best performance in FECT, as indicated by the F1 score.

**Table 5:** Summary of metrics evaluation for conventional methods performed by medical technologists

	Acc	Prec	Sen	Spec	F1
FECT					
• Medical Technologist A	94.61	63.49	66.67	96.88	65.04
• Medical Technologist B	94.49	67.39	51.67	97.97	58.49
MIF					
Medical Technologist A	95.22	65.63	52.50	98.13	58.33
Medical Technologist B	95.85	70.59	60.00	98.30	64.86

### Deep-learning-based evaluation

Confusion matrices were utilized to calculate the performance metrics—accuracy, precision, sensitivity, specificity, and F1 scores—providing insights into the diagnostic capabilities of artificial intelligence (AI)-based tools as scalable alternatives to conventional methods for IPI. Key evaluation metrics for class-wise prediction performance included precision, sensitivity, and F1 scores. The results (Tables 6 and 7) were derived from a default 0.4 non-maximum suppression (YOLO models) with a 50% threshold across all evaluated classes. For SL-based models, the object detection and classification approaches involved ResNet-50, YOLOv4-tiny, YOLOv7-tiny, and YOLOv8-m, while DINOv2 variants (DINOv2-base, DINOv2-small, and DINOv2-large) were employed for SSL.

**Table 6:** Class-wise prediction metrics of selected ResNet-50 and YOLO models

Class (species)	ResNet-50			YOLOv4-tiny		
	Prec	Sen	F1	Prec	Sen	F1
*A. lumbricoides* (decorticated)	46.70	73.61	57.14	97.74	75.00	84.87
*A. lumbricoides* (fertilized)	79.36	82.38	80.84	85.93	48.97	62.39
*A. lumbricoides* (unfertilized)	38.89	16.20	22.88	68.84	63.43	66.02
Hookworms	65.82	96.30	78.20	0.00	0.00	0.00
*Blastocystis* spp.	4.87	12.50	7.01	6.65	12.50	8.69
*E. coli*	24.32	2.08	3.84	67.41	42.13	51.85
*E. histolytica*	0.00	0.00	0.00	43.31	25.46	32.07
*E. nana*	5.58	8.33	6.69	8.35	6.77	7.48
*G. duodenalis*	9.18	5.56	6.92	30.77	0.62	1.21
*O. viverrini*	26.60	39.17	31.69	94.70	84.44	89.28
*S. stercoralis*	66.67	70.83	68.69	97.30	100.00	98.63
*Taenia* spp.	81.68	43.33	56.62	100.00	55.56	71.43
*T. trichiura*	38.64	15.74	22.37	96.41	74.54	84.07
*Toxocara* spp.	59.72	99.01	74.50	78.83	64.29	70.82

Class (species)	YOLOv7-tiny			YOLOv8-m		
	Prec	Sen	F1	Prec	Sen	F1
*A. lumbricoides* (decorticated)	48.98	75.00	59.26	68.63	72.92	70.71
*A. lumbricoides* (fertilized)	82.99	70.08	75.99	91.88	34.13	49.77
*A. lumbricoides* (unfertilized)	100.00	25.93	41.18	75.14	64.35	69.33
Hookworms	12.50	4.63	6.76	0.00	0.00	0.00
*Blastocystis* spp.	15.22	17.71	16.37	19.25	12.50	15.16
*E. coli*	89.73	54.63	67.91	79.58	69.44	74.17
*E. histolytica*	21.39	17.13	19.02	14.95	13.43	14.15
*E. nana*	32.65	5.56	9.50	57.14	4.86	8.96
*G. duodenalis*	32.61	4.63	8.11	80.00	63.58	70.85
*O. viverrini*	62.28	50.00	55.47	79.63	95.56	86.87
*S. stercoralis*	99.31	50.00	66.51	100.00	80.56	89.23
*Taenia* spp.	58.49	25.83	35.84	95.77	37.78	54.18
*T. trichiura*	74.51	17.59	28.46	50.81	57.87	54.11
*Toxocara* spp.	85.71	14.29	24.49	72.00	64.29	67.92

Notes: *Prec* precision, *Sen* sensitivity, *F1* F1 score

**Table 7:** Class-wise prediction metrics of DINOv2 variants

Class (species)	DINOv2-base			DINOv2-small			DINOv2-large		
	Prec	Sen	F1	Prec	Sen	F1	Prec	Sen	F1
*A. lumbricoides* (decorticated)	85.71	75.00	80.00	54.55	75.00	63.16	75.00	75.00	75.00
*A. lumbricoides* (fertilized)	96.55	77.78	86.15	88.89	66.67	76.19	96.97	88.89	92.75
*A. lumbricoides* (unfertilized)	100.00	66.67	80.00	50.00	33.33	40.00	100.00	83.33	90.91
Hookworms	0.00	0.00	0.00	30.77	50.00	38.10	100.00	12.50	22.22
*Blastocystis* spp.	16.67	50.00	25.00	5.00	12.50	7.14	5.26	12.50	7.41
*E. coli*	0.00	0.00	0.00	0.00	0.00	0.00	40.00	33.33	36.36
*E. histolytica*	0.00	0.00	0.00	0.00	0.00	0.00	0.00	0.00	0.00
*E. nana*	40.00	12.50	19.05	6.90	12.50	8.89	15.38	12.50	13.79
*G. duodenalis*	100.00	5.56	10.53	0.00	0.00	0.00	100.00	5.56	10.53
*O. viverrini*	33.33	80.00	47.06	40.00	20.00	26.67	100.00	90.00	94.74
*S. stercoralis*	83.33	62.50	71.43	77.78	87.50	82.35	100.00	100.00	100.00
*Taenia* spp.	50.00	20.00	28.57	33.33	20.00	25.00	64.29	90.00	75.00
*T. trichiura*	80.00	66.67	72.73	100.00	16.67	28.57	100.00	83.33	90.91
*Toxocara* spp.	81.82	64.29	72.00	75.00	64.29	69.23	100.00	64.29	78.26

Notes: *Prec* Precision, *Sen* sensitivity, *F1* F1 score

ResNet-50 achieved mixed results, with precision, sensitivity, and F1 scores above 59% for identifying fertilized *A. lumbricoides*, hookworms, *S. stercoralis*, *Taenia* spp., and *Toxocara* spp. However, it struggled with species such as *G. duodenalis*, *E. nana*, and *Blastocystis* spp., recording low metrics, and failed to classify *E. histolytica*. YOLOv4-tiny showed strong performance across 14 classes, particularly for *Taenia* spp., decorticated *A. lumbricoides*, *S. stercoralis*, *T. trichiura*, *O. viverrini*, and *Toxocara* spp., achieving at least 70% F1 scores due to distinctive morphological features. However, it encountered challenges with protozoa, showing lower F1 scores for species with shared characteristics, such as *Blastocystis* spp., *E. nana*, and *G. duodenalis*. YOLOv7-tiny exhibited slight variations in performance, particularly struggling with *E. nana*, *G. duodenalis*, and hookworms, which had the lowest F1 scores at 6.76%. In contrast, YOLOv8-m marked a significant improvement over previous models, achieving high metric values, especially for *S. stercoralis*, *O. viverrini*, *E. coli*, *G. duodenalis*, and decorticated *A. lumbricoides* at above 70%. However, despite its superior overall performance, YOLOv8-m also exhibited low efficacy in classifying *E. nana* and had null metrics for hookworms.

DINOv2-base balance computational efficiency and performance, showing high precision, sensitivity, and F1 scores for all *A. lumbricoides*, *T. trichiura*, *Toxocara* spp., and *S. stercoralis*. Although the model identified *G. duodenalis* with 100% precision, its low sensitivity resulted in a low overall performance. Further, lower metrics were recorded for *Taenia* spp., *Blastocystis* spp., and *E. nana*, and even null values for hookworms, *E. coli*, and *E. histolytica*. DINOv2-small focused on speed and efficiency, obtaining 100% precision for fertilized *A. lumbricoides*, *S. stercoralis*, *Toxocara* spp., and *T. trichiura*, but low sensitivity, thus low F1 scores. In addition, lower metrics were recorded for *E. nana* and *Blastocystis* spp., and null values for *E. coli*, *E. histolytica*, and *G. duodenalis*. Moreover, DINOv2-large offers the highest accuracy, demonstrating high metric values of at least 64% for *S. stercoralis*, *O. viverrini*, unfertilized *A. lumbricoides*, *T. trichiura*, and *Toxocara* spp. Among the high values recorded, the model still has difficulty predicting *E. histolytica* with null values, which was a consistent challenge for all the models.

**Fig 6.**
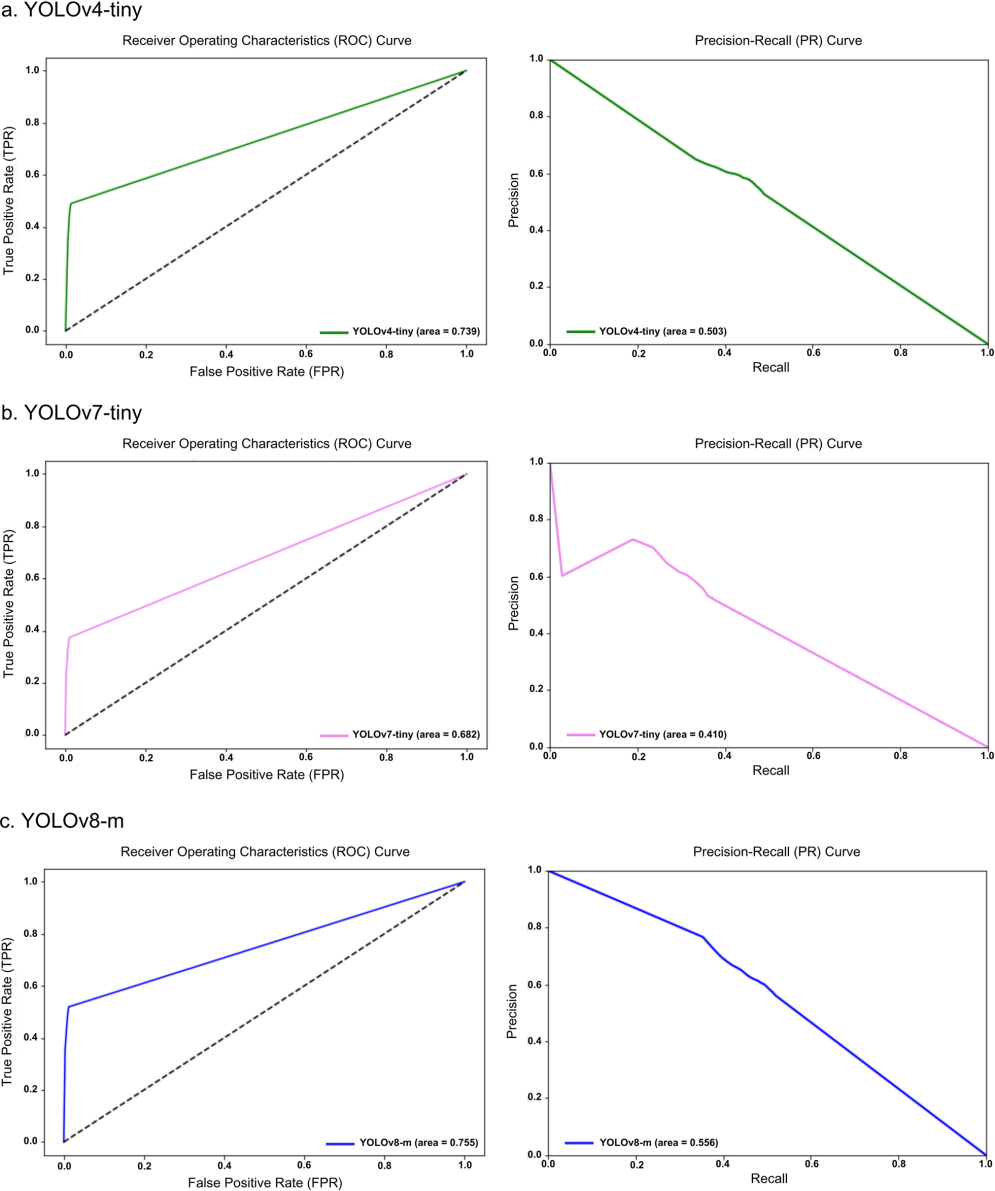

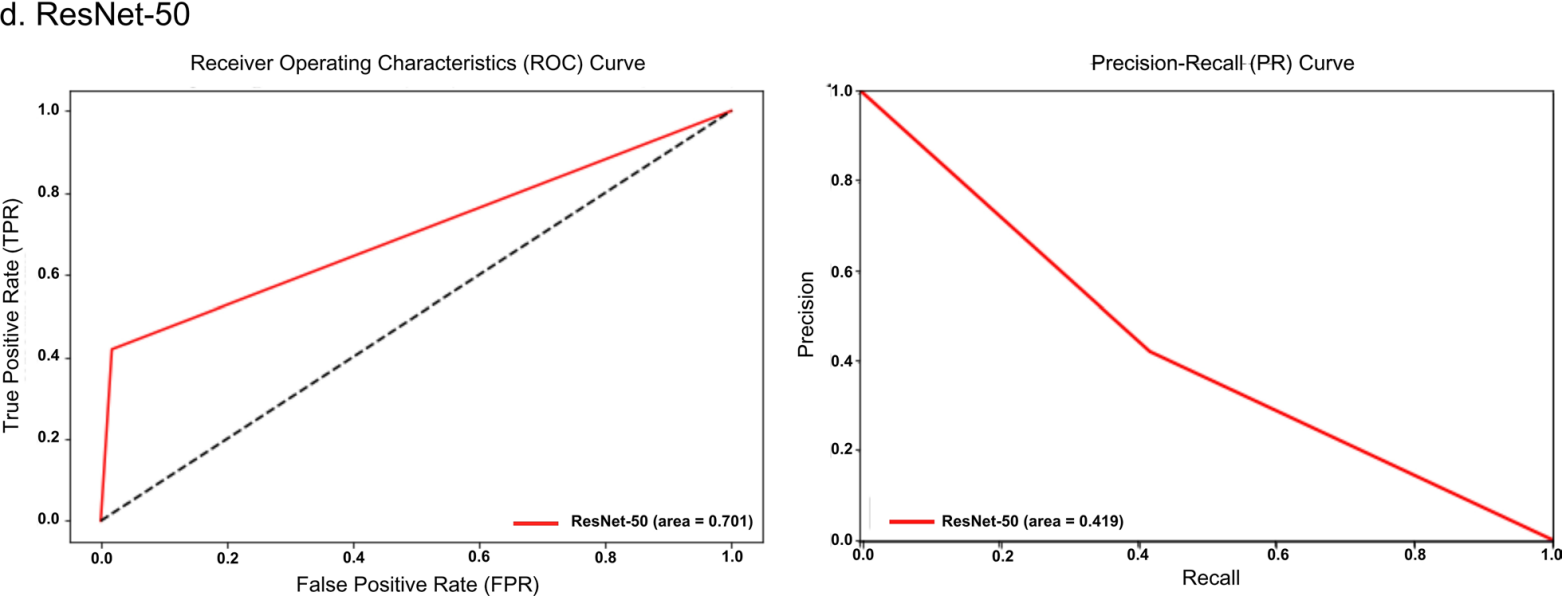
ROC and PR curves of SL-based models: (**a**) YOLOv4-tiny, (**b**) YOLOv7-tiny, (**c**) YOLOv8-m, and (**d**) ResNet-50

**Fig 7.**
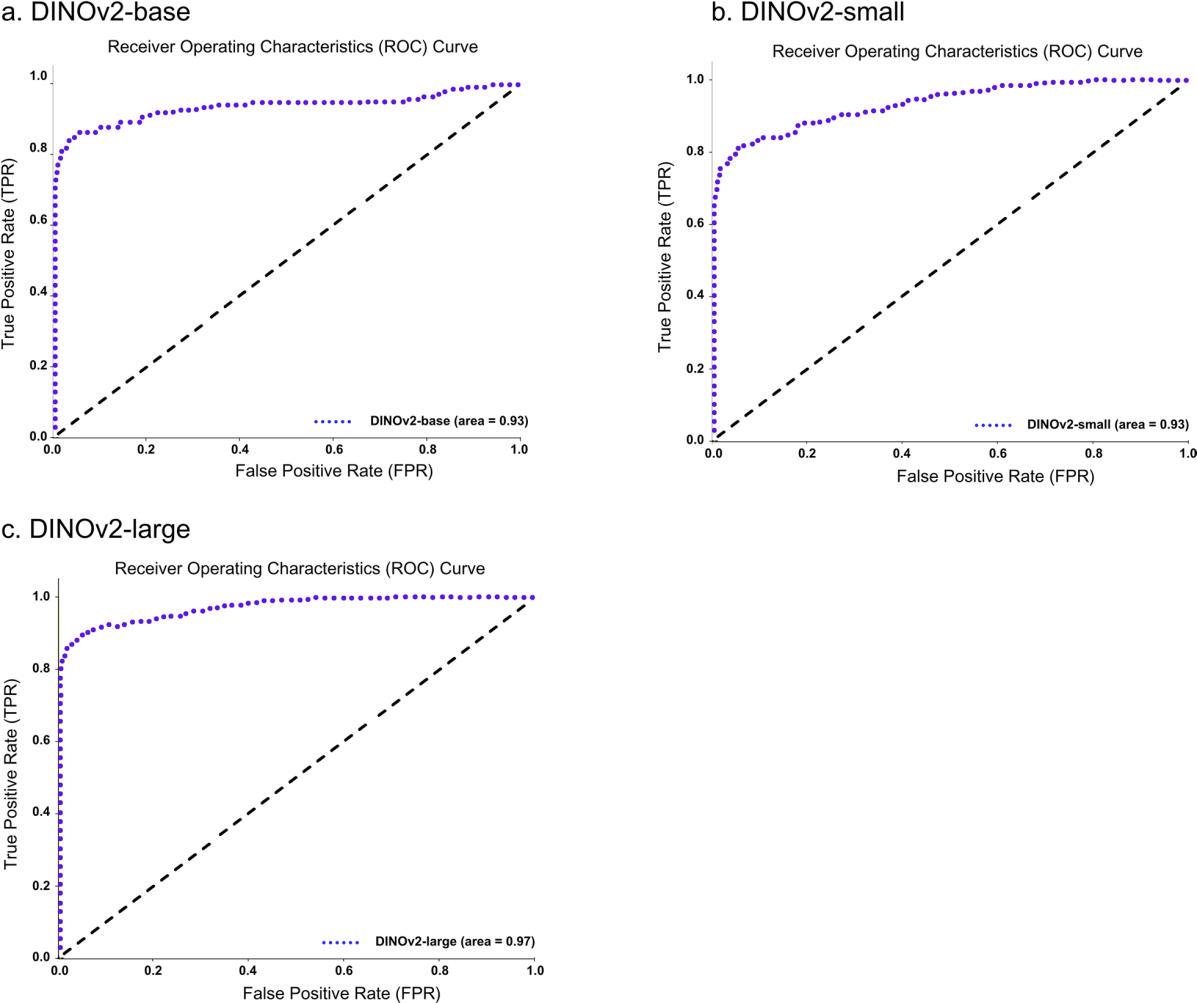
ROC curves and AUROC of SSL-based model: (**a**) DINOv2-base, (**b**) DINOv2-small, and (**c**) DINOv2-large

**Fig 8.**
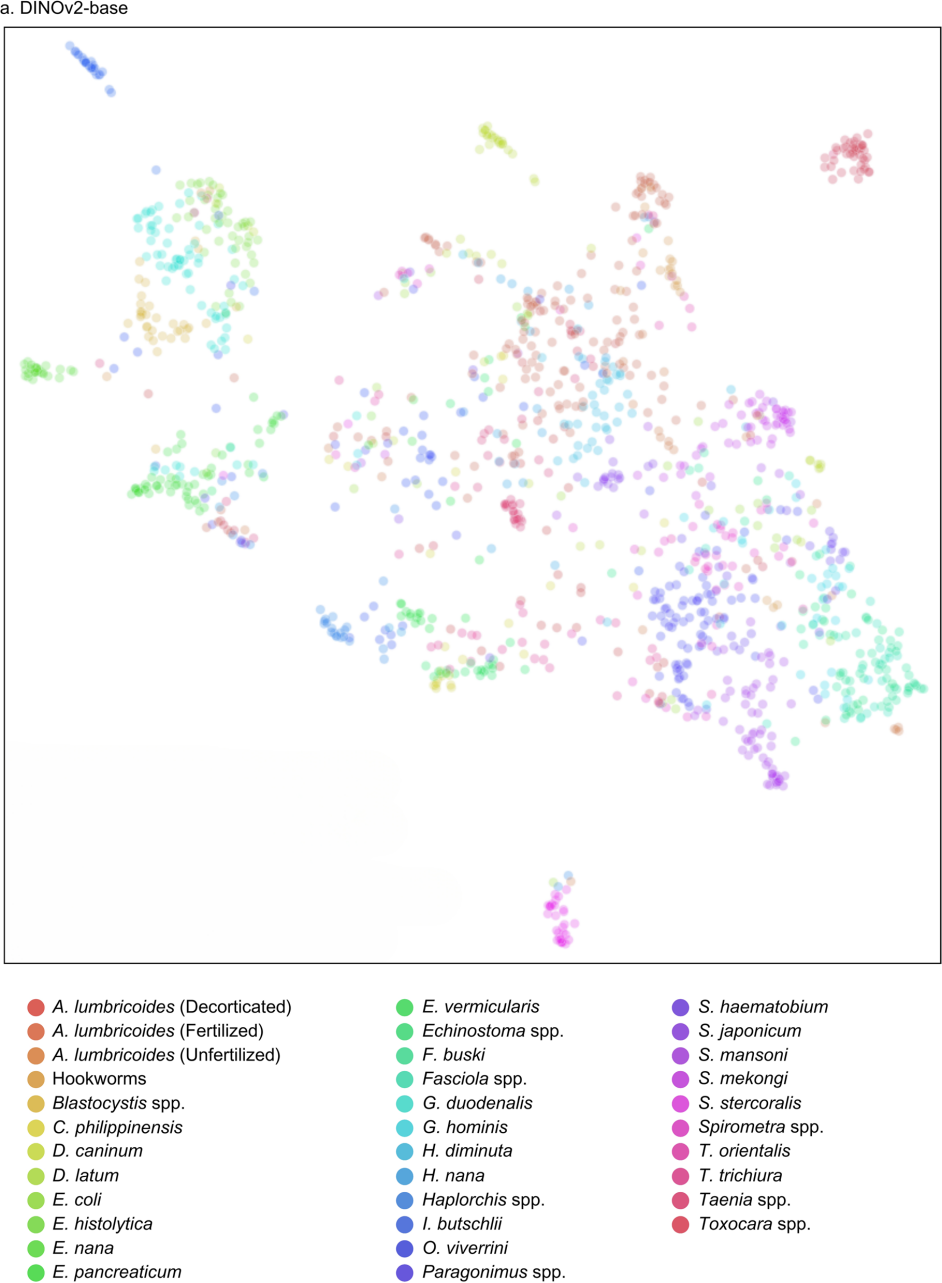

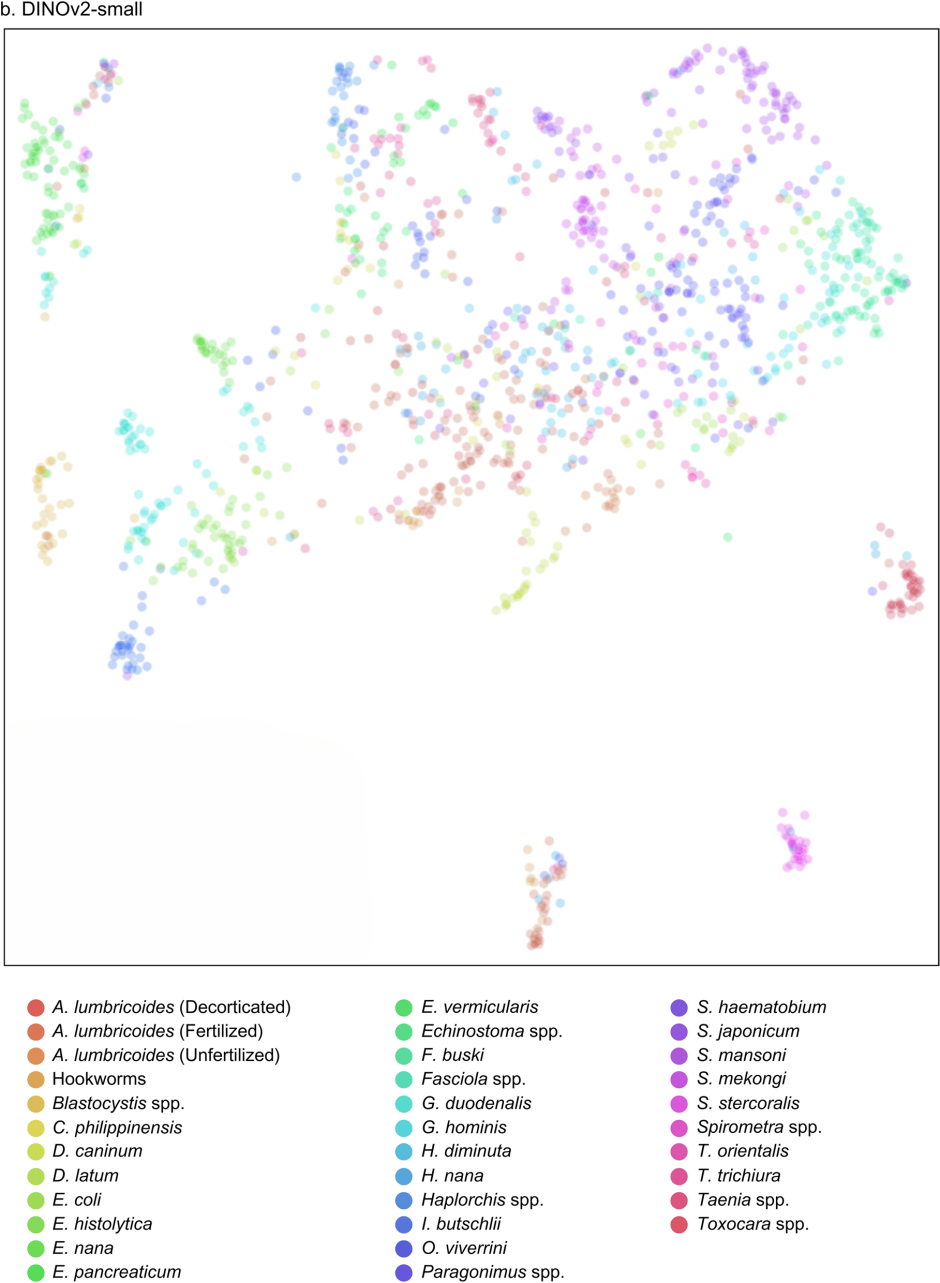

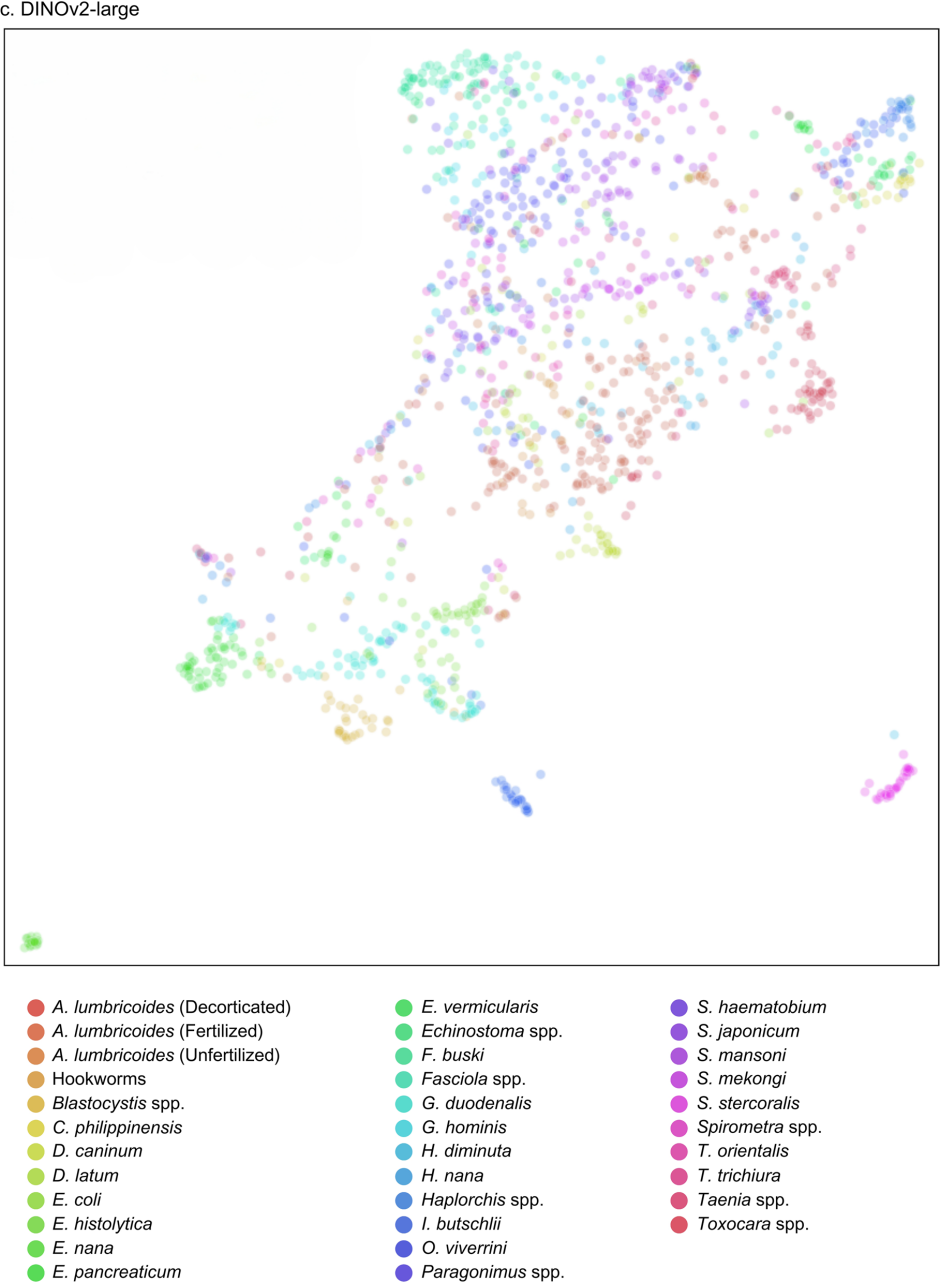
UMAP of SSL-based model showing cluster visualization by class: (**a**) DINOv2-base, (**b**) DINOv2-small, and (**c**) DINOv2-large

Additionally, an evaluation framework including accuracy, specificity, ROC, and PR curves with AUROC and AUPR values provided a comprehensive comparison of model performances against conventional stool examination methods by medical technologists, as summarized in Table 8. Notably, DINOv2-large produced exceptional results among the models evaluated. Among the SL-based models, YOLOv8-m exhibited the highest performance metrics, while ResNet-50 performed the least across all measures. The performance of both SL- and SSL-based models was competitive with that of employed medical technologists. Visual evaluations, including ROC and PR curves (Figs. 6, 7) and UMAP representations (Fig. 8), illustrated the models’ overall performance. YOLOv8-m achieved the highest AUROC and AUPR, demonstrating superior diagnostic capabilities despite the challenges of imbalanced datasets. Conversely, DINOv2-large recorded the highest AUROC among all models, with DINOv2-base and DINOv2-small slightly trailing but still outperforming ResNet-50 and YOLO. UMAP analysis showed that DINOv2-large effectively distinguished between different parasites, indicated by closely clustered groups and a robust representation of class predictions.

**Table 8:** Model-wise comparison of evaluation metrics among medical technologists and deep-learning-based models

	Acc	Prec	Sen	Spec	F1
Human experts (medical technologists)					
▪ FECT	94.55	65.44	59.17	97.43	61.77
▪ MIF	95.53	68.11	56.25	98.21	61.60
Supervised learning					
▪ YOLOv4-tiny	97.45	58.91	43.68	99.08	50.16
▪ YOLOv7-tiny	97.24	54.81	35.41	99.12	43.03
▪ YOLOv8-m	97.59	62.02	46.78	99.13	53.33
▪ ResNet-50	96.58	41.89	41.89	98.24	41.89
Self-supervised learning					
▪ DINOv2-base	98.71	82.30	71.71	99.53	76.64
▪ DINOv2-small	98.39	74.69	68.29	99.30	71.34
▪ DINOv2-large	98.93	84.52	78.00	99.57	81.13

Notes: *Acc* accuracy, *Prec* precision, *Sen* sensitivity, *Spec* specificity, *F1* F1 score

### Cohen’s kappa and Bland–Altman agreement measures

A comparison between human experts and deep/learning-based approaches was pivotal in pursuing advanced diagnostic accuracy for intestinal parasites. Two statistical methods—Cohen’s kappa coefficient and Bland–Altman analysis—evaluated the agreement in identifying various parasite classes. Cohen’s kappa (Table 9) assessed categorical agreement beyond chance, revealing a high level of consistency in classification decisions. Findings for the Kappa (*κ*) score (Table 10) revealed strong agreement (> 0.90), translating to 82–100% data reliability, with medical technologist B achieving a notable *κ* score of 0.9548 for human experts. SL models such as YOLOv7-tiny scored 0.9773, while SSL model DINOv2-large recorded the highest *κ* score of 0.9890. The significant *P*-value of < 0.0001 across categories rejected the null hypothesis, indicating a meaningful difference in diagnostic performance.

**Table 9:** Kappa score interpretation table

Kappa value (*κ*)	Level of agreement	% of data reliable
0–0.20	None	0–4
0.21–0.39	Minimal	5–15
0.40–0.59	Weak	16–35
0.60–0.79	Moderate	36–63
0.80–0.90	Substantial	64–81
> 0.90	Strong	82–100

**Table 10:** Kappa evaluation analyzed through RStudio

Model	*κ*	SE	*Z*-value	*P*-value
Human experts (medical technologists)				
▪ FECT A	0.9425	0.0085	110.5965	< 0.0001
▪ FECT B	0.9411	0.0086	109.1085	< 0.0001
▪ MIF A	0.9479	0.0093	102.1355	< 0.0001
▪ MIF B	0.9548	0.0087	110.1755	< 0.0001
Supervised learning				
▪ YOLOv4-tiny	0.9737	0.0004	2680.4045	< 0.0001
▪ YOLOv7-tiny	0.9773	0.0003	3567.2529	< 0.0001
▪ YOLOv8-m	0.9752	0.0003	2762.0828	< 0.0001
▪ ResNet-50	0.9648	0.0004	2305.7730	< 0.0001
Self-supervised learning				
▪ DINOv2-base	0.9868	0.0010	928.8853	< 0.0001
▪ DINOv2-small	0.9834	0.0011	827.7671	< 0.0001
▪ DINOv2-large	0.9890	0.0009	1020.7787	< 0.0001

Notes: *κ* Kappa score, *A* Medical Technologist 1, *B* Medical Technologist 2, *SE* standard error

**Fig 9.**
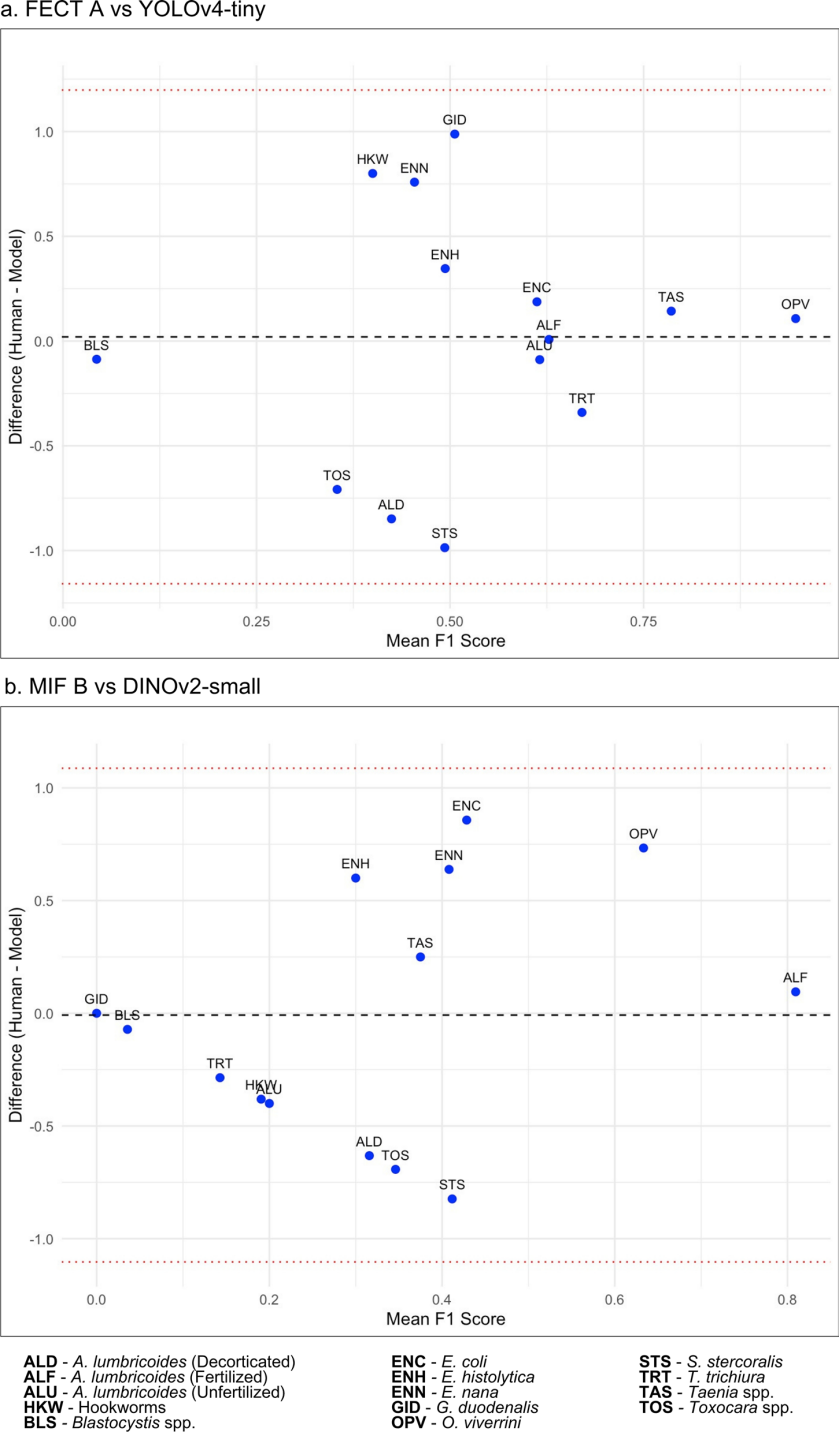

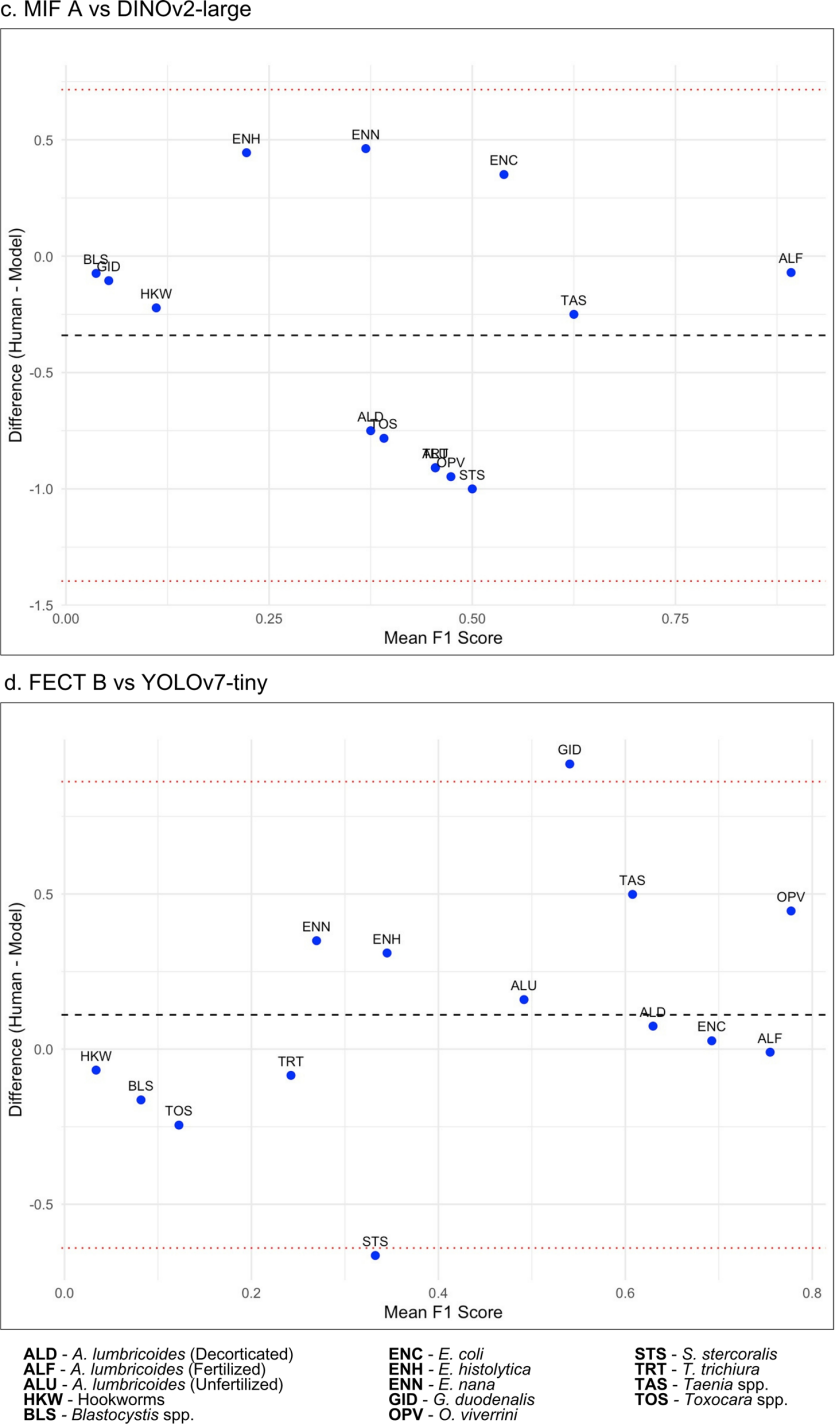
Bland–Altman plots of highlighted agreement between human experts and deep-learning-based models per parasite class: (**a**) FECT A versus YOLOv4-tiny, (**b**) MIF B versus DINOv2-small, (**c**) MIF A versus DINOv2-large, and (**d**) FECT B versus YOLOv7-tiny

Meanwhile, the Bland–Altman analysis (Table 11) showed the summary of agreements while the plots (Fig. 9) visually assessed agreement in F1 scores between raters for each parasite class. FECT Med Tech A showed minor mean differences across models, with the ResNet-50 and DINOv2-small having the most considerable mean differences (0.1709 and 0.2081), respectively, while DINOv2-large had a slight negative mean difference (−0.0223). In contrast, FECT Med Tech B displayed a mix of positive and negative mean differences, notably with YOLOv7-tiny (0.1105) exceeding 14% outside the limits of agreement. All models exhibited negative mean differences for MIF Med Tech A and B, with DINOv2-large showing the most significant deviation (−0.3402). Overall, the analysis highlighted varying degrees of agreement between human experts and models, indicating that while some models align closely with human assessments, others demonstrate considerable discrepancies (Table 11).

**Table 11:** Bland–Altman summary of agreements using F1 scores between human experts and deep-learning-based models in parasite classification

Human experts	Deep-learning-based model	Mean Diff	SD Diff	% Outside LoA
FECT Med Tech A	ResNet_50	0.1709	0.5595	–
FECT Med Tech A	YOLOv4_tiny	0.0199	0.6012	–
FECT Med Tech A	YOLOv7_tiny	0.1727	0.4924	–
FECT Med Tech A	YOLOv8_m	0.0223	0.5174	–
FECT Med Tech A	DINOv2_base	0.1346	0.6044	–
FECT Med Tech A	DINOv2_small	0.2081	0.5966	–
FECT Med Tech A	DINOv2_large	−0.0223	0.6036	–
FECT Med Tech B	ResNet_50	0.1087	0.5452	–
FECT Med Tech B	YOLOv4_tiny	−0.0423	0.4911	7.1429
FECT Med Tech B	YOLOv7_tiny	0.1105	0.3834	14.2857
FECT Med Tech B	YOLOv8_m	−0.0399	0.3835	7.1429
FECT Med Tech B	DINOv2_base	0.0724	0.5046	–
FECT Med Tech B	DINOv2_small	0.1459	0.5427	–
FECT Med Tech B	DINOv2_large	−0.0845	0.5188	–
MIF Med Tech A	ResNet_50	−0.1470	0.4658	–
MIF Med Tech A	YOLOv4_tiny	−0.2980	0.5060	–
MIF Med Tech A	YOLOv7_tiny	−0.1452	0.3402	–
MIF Med Tech A	YOLOv8_m	−0.2956	0.4861	–
MIF Med Tech A	DINOv2_base	−0.1833	0.5035	–
MIF Med Tech A	DINOv2_small	−0.1098	0.4716	–
MIF Med Tech A	DINOv2_large	−0.3402	0.5387	–
MIF Med Tech B	ResNet_50	−0.0452	0.5539	–
MIF Med Tech B	YOLOv4_tiny	−0.1962	0.5216	–
MIF Med Tech B	YOLOv7_tiny	−0.0434	0.3866	–
MIF Med Tech B	YOLOv8_m	−0.1938	0.5045	–
MIF Med Tech B	DINOv2_base	−0.0814	0.5710	–
MIF Med Tech B	DINOv2_small	−0.0080	0.5588	–
MIF Med Tech B	DINOv2_large	−0.2384	0.5629	–

Notes: *Mean Diff* mean difference, *SD Diff* standard deviation of the differences, *% Outside LoA* percentage outside the limits of agreement, *Med Tech* medical technologist

## Discussion

This study explored the capabilities of models such as ResNet-50, YOLOv4-tiny, YOLOv7-tiny, YOLOv8-m, and DINOv2 compared with conventional methods such as FECT and MIF techniques performed by medical technologists. Compressed versions of the selected models were used to accommodate the limited computing power of the hardware, thus the versions -tiny, -m, base, small, and large. Stool examination traditionally depends on the analysts’ expertise to differentiate parasites from artifacts and classify them into species. While performance metrics among the medical technologists were similar, human expertise remains crucial for determining overall effectiveness. Parasites exhibit various developmental stages with distinct morphologies, demanding keen attention to diagnostic stages for accurate identification. For instance, decorticated and unfertilized *A. lumbricoides* may indicate past infections despite their inability to cause new infections. Recently, researchers have introduced artificial intelligence into parasitological diagnostics via image analysis, aiming to enhance conventional coprological examinations. Deep learning approaches, powered by advanced algorithms, offer faster and more effective analyses due to superior computational capacities [34, 35].

ResNet-50 is a CNN that addresses deep network training challenges in image classification through bottleneck residual blocks and skip connections, enhancing information flow and accuracy. In contrast, compared with earlier versions, YOLOv4 uses the CSPDarknet53 backbone for improved learning, incorporating a composite loss function that combines bounding box regression, confidence, and classification losses for more efficient object predictions [36]. YOLOv7 boosts speed and accuracy with the E-ELAN computational block, and YOLOv8 directly predicts object centers, enabling faster NMS and advanced convolution methods [37, 38]. While YOLOv4 and YOLOv8 are efficient models, they require more memory and computational resources during training than YOLOv4-tiny. In deployment scenarios, YOLOv8 variants can match or surpass the efficiency of the complete YOLOv4 model, making the choice between YOLOv4 and YOLOv8 dependent on application needs. For those with strict hardware constraints, YOLOv4-tiny is the preferred option. However, if high detection accuracy is required and hardware upgrades are possible, YOLOv8 offers a better balance between performance and resource use. In addition, YOLO excels at detecting parasites with distinct morphological features in complex environments, while ResNet-50 primarily focuses on image classification, which limits its effectiveness in such contexts.

This study advances the application of automation in parasitology by utilizing advanced object detection architectures such as YOLOv8-m and DINOv2-large, which outperform traditional CNNs in species recognition compared with previous research focused on helminth eggs and protozoan cysts. YOLOv8-m stood out among SL models for its superior F1 scores across species with distinct morphological features such as *S. stercoralis*, *E. coli*, and *O. viverrini*, and notably overcame challenges in earlier YOLO versions, which exhibited reduced sensitivity for morphologically ambiguous or underrepresented species. These findings align with similar studies in object detection for parasitic disease, where model refinement significantly improved detection performance across heterogeneous datasets [39, 40]. The SSL-based DINOv2-large model demonstrated the highest overall performance, validating that transformer architectures surpass traditional CNNs when pretrained and fine-tuned. Its capability to extract robust features from images without requiring extensive labeled datasets is particularly beneficial in medical fields with limited annotations [41, 42]. This model’s high *κ* score (0.9890) and minimal mean difference in Bland–Altman plots signify strong alignment with expert evaluations, outperforming ResNet-50, and in some cases, even human experts. These findings align with the growing body of literature affirming the efficacy of vision transformers and SSL models in medical imaging, where they consistently excel in feature extraction and classification for complex tasks [43].

Despite these advantages, several limitations were still observed. Across both SL and SSL models, the identification of protozoan species such as *Blastocystis spp.*, *E. nana*, and *G. duodenalis* consistently yielded lower F1 scores, underscoring ongoing challenges in the automated detection of morphologically similar or low-contrast parasite forms, which are also prone to human misidentification [44]. Moreover, despite efforts to ensure high-quality annotations, inconsistencies in bounding box precision and labeling were still noted, such as for hookworms in cases predicted by the models as *A. lumbricoides*, *T. orientalis*, and *E. vermicularis.*. ResNet-50, although historically popular in image classification tasks, exhibited the poorest performance across all metrics, which reaffirms its limitations in fine-grained classification tasks requiring the detection of subtle morphological differences and its inferior capacity relative to modern object detectors and transformer-based SSL models. Another key insight is that while high precision and sensitivity were recorded for several classes, class imbalance and false negatives remained significant obstacles, especially in underrepresented classes. Underrepresentation in training datasets often leads to poor model generalization or class bias, and such disparities can lead to clinically significant errors, especially when critical infections are overlooked [45]. Additionally, despite the observed high accuracy and kappa agreement values, discrepancies in Bland–Altman plots, particularly for MIF technique comparisons, indicated that human-model variability remains nontrivial, especially when different staining or fixation methods influence image features [46, 47]. Furthermore, comparing observation with the time consumed from sampling or preparation to identification of parasites showed that using a deep-learning-based approach takes time during model training. SL models took at least 48 h and ResNet-50 were the longest at almost 120 h, while DINOv2 had an improvement at only 24 h. However, applying the model in the identifications is much faster than that of human experts. For instance, an analyst prepares each slide of stool samples and examines them under the microscope with the help of pertinent references for identification, or it is best when considered an expert, depending on skill and experience. Meanwhile, parasite identification and generating reports can be done in minutes through an automated approach, especially when microscopes have already been customized to screen prepared slides automatically [48].

These findings suggest that deep learning models—particularly YOLOv8-m and DINOv2-large—can aid or augment diagnostic workflows in stool microscopy, especially in low-resource settings where expert microscopists are limited. However, a hybrid approach that combines expert validation with model predictions is crucial to reduce false positives and negatives. Future enhancements through active learning, synthetic data augmentation, comprehensive dataset databases, and attention-based interpretability tools could further improve clinical application performance and trust [49]. Ultimately, this study demonstrates that state-of-the-art deep learning models, especially those based on SSL architectures, show substantial promise for automated parasite detection. However, continued refinements and better species-level annotation will be essential for real-world clinical integration.

## Conclusions

The study presented a pivotal evaluation of a deep-learning-based approach for identifying intestinal parasites, demonstrated by each selected model’s metric performances through *κ* scores for model-wise comparison and Bland–Altman analysis to visualize the agreement between the models and human experts who performed conventional methods in the identification of intestinal parasites from stool samples. The assessment of 14 different intestinal parasites using state-of-the-art models marks a critical step toward improving diagnostic precision, efficiency, cost-effectiveness, and quality that set the stage for future parasite detection and identification breakthroughs. It also emphasizes the significant shift toward automation in parasitology, showcasing the potential of automated detection systems to deliver more reliable and robust diagnostic outcomes for IPI on notable improvement over conventional techniques. Leveraging automation through SL- and SSL-based algorithms enables rapid and reliable identification of parasites in stool samples, leading to the timely diagnosis and treatment of infections, especially in remote and resource-limited settings. However, the effectiveness of these automated techniques depends heavily on the quality of training data that might influence the reliability of the results, such as the quantity of training dataset images, the inclusion of null images, and the allocation of a portion of the dataset for validation that could help the model become more efficient and improve the robustness of detection for real-world applications. Additionally, challenges such as interpretability and over-reliance must be carefully managed to ensure the systems are reliable and ethically deployed. Future advancements in data diversity, algorithm design, and transparency are critical to further improving the robustness and trustworthiness of these automated detection systems, ensuring they are effectively integrated into diagnostic practices.

## Data Availability

The datasets used in this study are available in the figshare repository [https://figshare.com/projects/Performance_Validation_of_Deep_Learning-based_Approach_in_Stool_Examination/234128]. All data generated are included in this article and its supplementary information files.

## Abbreviations

AUPR: Area under the precision-recall curve
AUROC: Area under the receiver operating characteristic
CNN: Convolutional neural network
DINO: DIstillation of knowledge with NO labels
FECT: Formalin-ethyl acetate concentration technique
IPI: Intestinal parasitic infection
MIF: Merthiolate-iodine-formalin
ResNet: Residual network
SL: Supervised learning
SSL: Self-supervised learning
UMAP: Uniform manifold approximation and projection
ViT: Vision transformers
YOLO: You only look once
